# Broader-species receptor binding and structural bases of Omicron SARS-CoV-2 to both mouse and palm-civet ACE2s

**DOI:** 10.1038/s41421-022-00431-0

**Published:** 2022-07-12

**Authors:** Linjie Li, Pu Han, Baihan Huang, Yufeng Xie, Weiwei Li, Di Zhang, Pengcheng Han, Zepeng Xu, Bin Bai, Jingya Zhou, Xinrui Kang, Xiaomei Li, Anqi Zheng, Rong Zhang, Shitong Qiao, Xin Zhao, Jianxun Qi, Qihui Wang, Kefang Liu, George Fu Gao

**Affiliations:** 1 CAS Key Laboratory of Pathogen Microbiology and Immunology, Institute of Microbiology, Chinese Academy of Sciences, Beijing, China; 2 University of Chinese Academy of Sciences, Beijing, China; 3 Department of Basic Medical Sciences, School of Medicine, Tsinghua University, Beijing, China; 4 Faculty of Health Sciences, University of Macau, Macau, Macau SAR China; 5 School of Medicine, Zhongda Hospital, Southeast University, Nanjing, Jiangsu China; 6 Cryo-EM Center, Shanxi Academy of Advanced Research and Innovation, Taiyuan, Shanxi China; 7 State Key Laboratory for Conservation and Utilization of Subtropical Agro-Bioresources, Guangxi University, Nanning, Guangxi China

**Keywords:** X-ray crystallography, Electron microscopy

## Abstract

The Omicron variant of SARS-CoV-2 carries multiple unusual mutations, particularly in the receptor-binding domain (RBD) of the spike (S) protein. Moreover, host-adapting mutations, such as residues 493, 498, and 501, were also observed in the Omicron RBD, which indicates that it is necessary to evaluate the interspecies transmission risk of the Omicron variant. Herein, we evaluated the interspecies recognition of the Omicron BA.1 and Delta RBDs by 27 ACE2 orthologs, including humans. We found that Omicron BA.1 expanded its receptor binding spectra to palm-civet, rodents, more bats (least horseshoe bat and greater horseshoe bat) and lesser hedgehog tenrec. Additionally, we determined the cryo-electron microscopy (cryo-EM) structure of the Omicron BA.1 S protein complexed with mouse ACE2 (mACE2) and the crystal structure of Omicron RBD complexed with palm-civet ACE2 (cvACE2). Several key residues for the host range have been identified. These results suggest that surveillance should be enhanced on the Omicron variant for its broader-species receptor binding to prevent spillover and expansion of reservoir hosts for a prolonged pandemic.

## Introduction

Since its outbreak, SARS-CoV-2 has shown marked mutational diversity, resulting in the emergence of multiple variants^1,2^. The SARS-CoV-2 variant Omicron BA.1 (B.1.1.529) was first reported to the World Health Organization on November 24, 2021, and classified as a variant of concern (VOC) two days later^1^. The emergence of Omicron triggered another wave of infection that spread to six continents within a week, resulting in global panic and concern^3,4^.

Human-to-animal transmission of SARS-CoV-2 has been reported in several countries^5,6^. Naturally-infected animals include cats, dogs, minks, tigers, African lions, white-tailed deer, ferrets, pumas, gorillas and snow leopards, as well as hippopotamus, in the most recently reported cases of this type of transmission (https://www.bbc.com/news/world-europe-59516896)^5,7–11^. Particularly, mink-to-human transmission has also been reported to cause community transmission^9^. In addition to natural infections, experimental infections have also identified several animals (e.g., rabbit, pig, and fox) as potential susceptible hosts for SARS-CoV-2^5^. Molecular and cellular assays have further demonstrated a broad receptor-binding spectra covering domestic animals, pets, livestock, and animals commonly found in zoos and aquaria, in addition to many wild animals, including several species of bats^12–14^.

Genomic analysis shows that Omicron BA.1 carries an unusually large number of mutations, particularly in the S protein^15,16^. There are 15 amino acid (AA) substitutions in the receptor binding domain (RBD) of the S protein, nine of which are located at the binding interface of the SARS-CoV-2 RBD and human ACE2 (hACE2) receptor^17^, namely K417N, G446S, S477N, E484A, Q493R, G496S, Q498R, N501Y, and Y505H. K417N, E484A, and N501Y have been identified as key mutations in previous VOCs^18^ whereas residues at sites 493, 498, and 501 are known to be determinants of the host range^5,19^. Considering that the Omicron BA.1 variant contains mutations at all host range-determining sites as we predicted, evaluating the potential host range of the variant is vital for the provision of guidance for animal surveillance.

Since the outbreak of the Omicron BA.1 variant, investigations on the structural basis and host range of this variant have been conducted^20^. In addition to the crystal and cryo-EM structures of Omicron BA.1S protein complexed with hACE2^20–22^, the structures of Omicron S protein alone, and in complex with neutralizing antibodies have also been determined^22,23^. The results showed that these heavy mutations gave rise to new characteristics, while mutations located on the ACE2-binding interface combined to exert a compensation effect, resulting in a binding affinity of Omicron RBD comparable to that of hACE2^20^. Moreover, in the structure of the S protein, some mutations (e.g., N746K, T547K, and N856K) have been found to introduce interprotomer electrostatic contacts and stabilize the conformation of the S protein^22^. However, the structural basis of receptor recognition by the Omicron variant in its potential animal host has yet to be elucidated.

Although the origin of Omicron BA.1 remains mystery, three hypotheses have been proposed^24^. One possibility is that Omicron or its ancestral variant has been undetected in humans for a long time. Another hypothesis postulates that Omicron evolved in immunocompromised people^24,25^. Thirdly, Omicron may originate from adaptations in animal reservoirs. Wei et al. reported that the molecular spectrum of mutations acquired by the progenitor of Omicron was significantly different from the spectrum of other SARS-CoV-2 variants that evolved in human patients, but resembled the spectra associated with virus evolution in a mouse cellular environment^26^. They speculated that the progenitor virus of Omicron was transmitted from humans to mice, rapidly mutated to adapt to the host, and then jumped back to humans. Consistent with their hypothesis, direct contact transmission of the SARS-CoV-2 variant B.1.351 in wild-type mice has been reported to lead to significant pathological changes^27^. Recently, 11 hamsters from a pet store in Hong Kong, China, were reported to be infected with the Delta variant (https://www.scmp.com/news/hong-kong/health-environment/article/3164000/hong-kong-hamster-cull-top-covid-19-expert). The transmission of SARS-CoV-2 from humans to mice drives viral evolution and adaption to the hosts.

In this study, the receptor binding capability of the Omicron BA.1 and Delta variants to 27 species were compared, including humans, typical domestic animals, and wildlife animals. Omicron BA.1 was found to display a broader host range than the prototype, whereas the Delta variant showed no alteration. We further determined the cryo-EM structure of the Omicron BA.1 S protein in complex with mACE2, as well as the crystal structure of Omicron RBD complexed with cvACE2, and elucidated the molecular mechanism underlying the receptor-binding spectra expansion of Omicron BA.1. Our work sheds light on the potential mechanism for host adaption of the Omicron BA.1 variant and highlights the necessity of continuous surveillance of susceptible species to avoid further spillover of SARS-CoV-2.

## Results

### RBD mutations of the Omicron BA.1 variant

Fifteen substitutions were observed in the RBD of the Omicron BA.1 strain, of which nine were located on the hACE2-binding surface^20^. To investigate their potential host range, RBD sequence alignment with the prototype SARS-CoV-2 RBD and Omicron RBD was conducted to highlight their locations (Supplementary Fig. S1a, b). Prototype, Delta, Omicron, and three close relatives of SARS-CoV-2 (bat-origin RaTG13, pangolin-origin GD/1/2019 and GX/P2V/2017) were included for comparison. Omicron BA.1 shares three important substitutions observed in previous VOCs, namely K417N and N501Y, whose effects on hACE2 binding have been extensively explored^28^. Notably, site 501 has also been reported to have a host range determinant^5^. Substitutions on the other two host-range-determining sites, 493 and 498, were also observed in Omicron BA.1 RBD. Q498R has been reported to emerge epistatically to N501Y and to markedly increase hACE2-binding affinity^29^. Q493R emerges after treatment with monoclonal antibodies and is associated with immune escape^30,31^. A structural analysis of hACE2 in complex with the Omicron BA.1 Spike (S) protein revealed that Omicron BA.1 R493 replaces the hydrogen bond (H-bond) between the ancestral Q493 and E35 on hACE2 with a salt bridge, and that Omicron BA.1 R498 forms an additional salt bridge with D38 in hACE2^21^. These two substitutions may compensate for the sabotaging effect of K417N on hACE2 binding.

### The receptor binding spectra of the Omicron variant is expanded

Receptor binding is the key step for SARS-CoV-2 infection. To evaluate whether the host ranges of the Delta and Omicron BA.1 variants were altered, the receptor binding characteristics of their RBD were evaluated using flow cytometry and surface plasmon resonance (SPR) assays. Additionally, we tested ACE2 proteins from 27 species (including humans) belonging to nine orders: Primates (human and monkey), Lagomorpha (rabbit), Rodentia (mouse, rat, and guinea pig), Pholidota (Malayan pangolin), Carnivora (cat, palm-civet, fox, dog, raccoon dog, and mink), Perissodactyla (horse), Artiodactyla (pig, wild Bactrian camel, alpaca, bovine, goat, and sheep), Chiroptera (intermediate horseshoe bat, least horseshoe bat, little brown bat, fulvous fruit bat, greater horseshoe bat and big-eared horseshoe bat) and Afrotheria (lesser hedgehog tenrec).

**Fig. 1:**
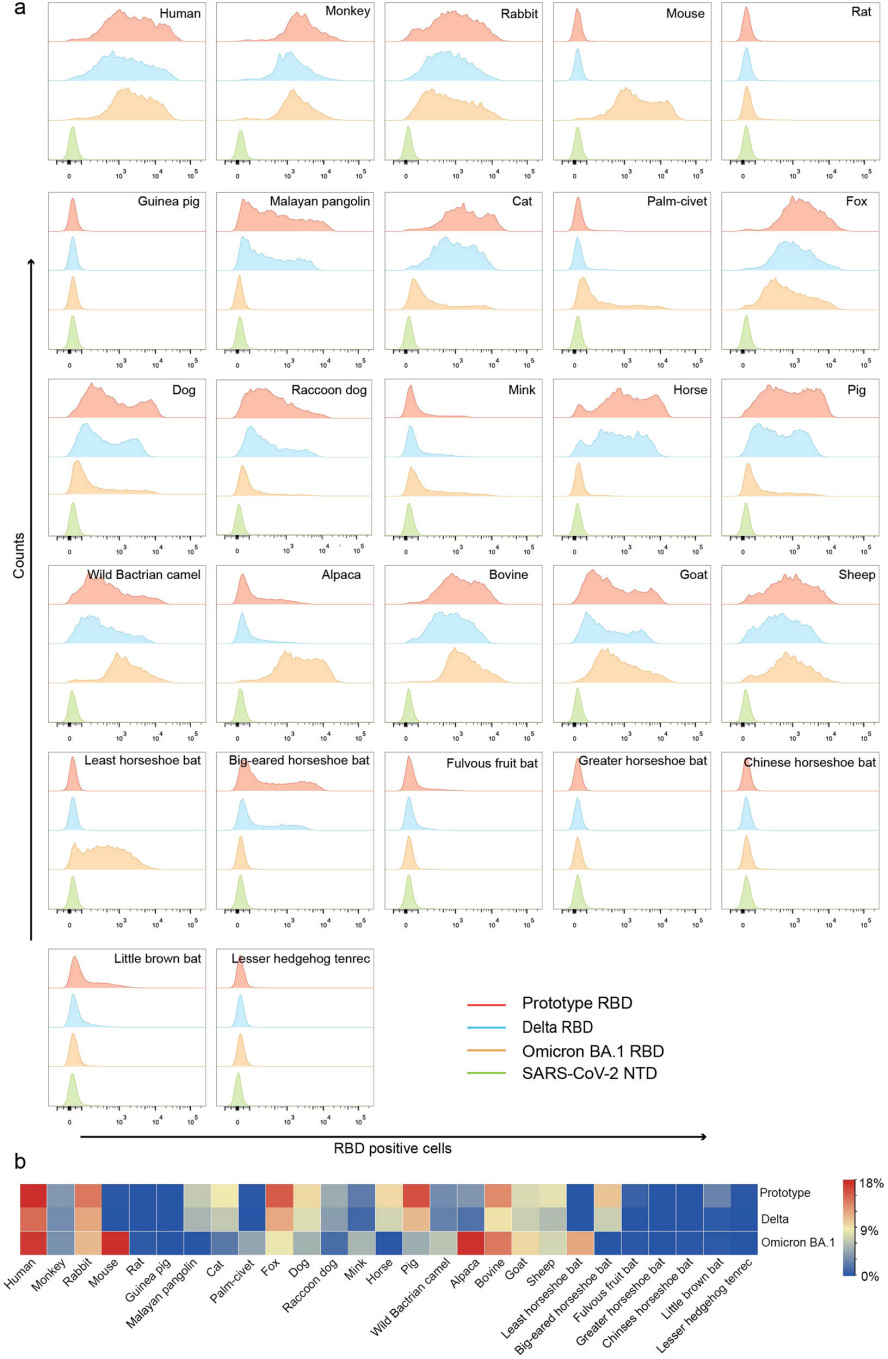
Flow cytometric characterization of the binding between ACE2s and prototype SARS-CoV-2 RBD, Delta RBD, and Omicron BA.1 RBD. **a** BHK-21 cells expressing eGFP-fused ACE2s were incubated with the indicated His-tagged proteins (prototype RBD, Delta RBD, Omicron BA.1 RBD, and SARS-CoV-2 NTD). An anti-His/Allophycocyanin (APC) antibody was used to detect His-tagged proteins. The SARS-CoV-2 NTD was used as a negative control. **b** The positive rate of prototype, Delta and Omicron RBDs for different ACE2 orthologs were presented as a heatmap according to the indicated color code.

Flow cytometry was used to test the binding of prototype, Delta and Omicron BA.1 RBDs to eGFP-fused ACE2s expressed on the cell surface. The Delta RBD displayed identical receptor binding spectra as the prototype RBD (Fig. 1), which was described in our previous work^13,32^. Meanwhile, Omicron BA.1 demonstrated a varied binding pattern. Briefly, monkey, rabbit, cat, fox, dog, raccoon dog, mink, pig, wild Bactrian camel, alpaca, bovine, goat, and sheep ACE2 orthologs were bound to all three RBDs tested using the flow cytometry assay. In addition, the Omicron BA.1 RBD demonstrated a broad binding capacity to mouse, palm-civet and least horseshoe bat ACE2s, whereas binding to rat, guinea pig, Malayan pangolin, horse, big-eared horseshoe bat, fulvous fruit bat, greater horseshoe bat, Chinese horseshoe bat, little brown bat and lesser hedgehog tenrec ACE2 orthologs could not be detected by flow cytometry (Fig. 1).

**Fig. 2:**
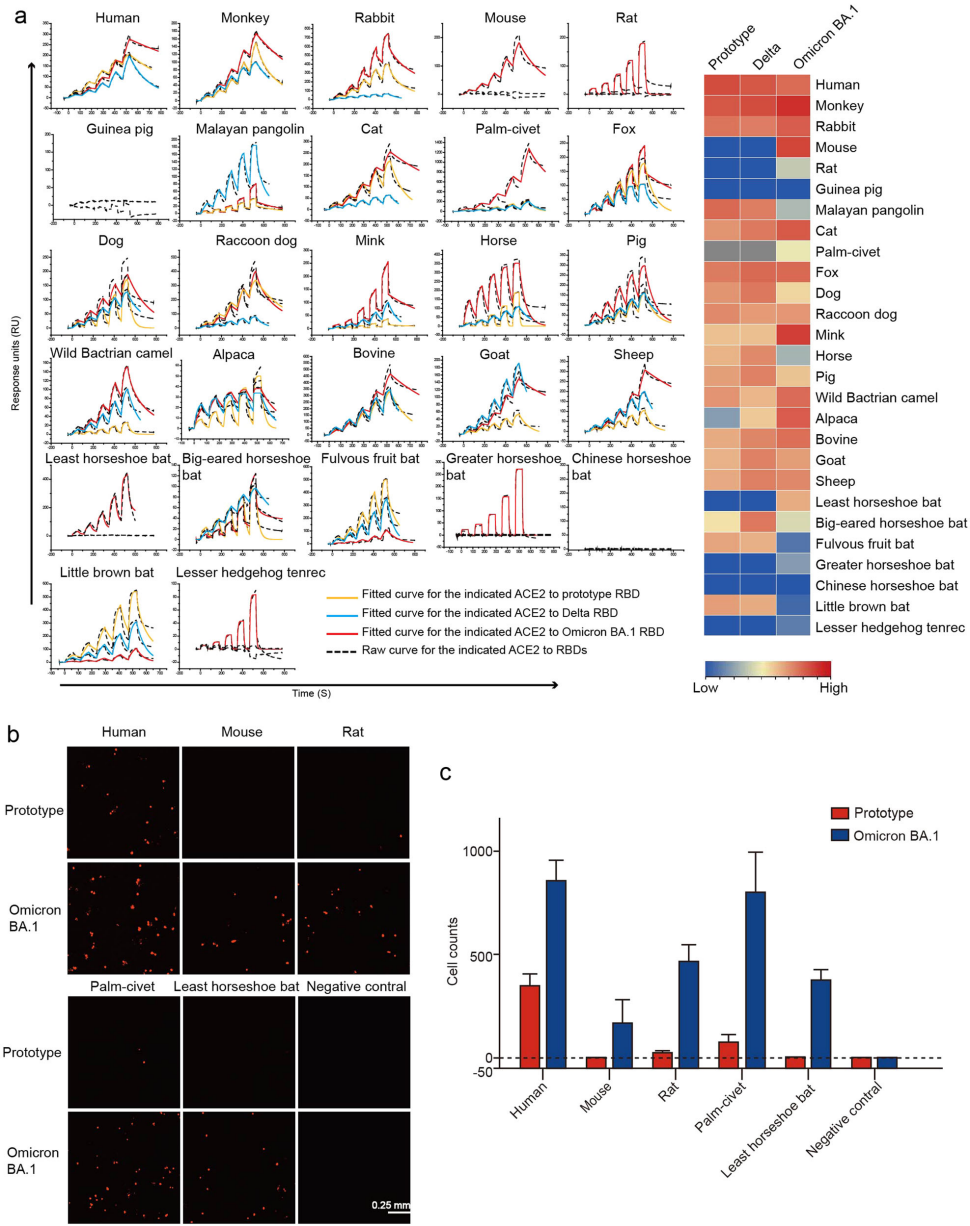
SPR characterization of the binding between ACE2s and SARS-CoV-2 prototype RBD, Delta RBD or Omicron BA.1 RBD. **a** ACE2s with a mouse Fc (mFc) tag were immobilized on a CM5 chip. SPR characterizations of the binding affinity between the prototype RBD (yellow curve), Delta RBD (blue curve), or Omicron BA.1 RBD (red curve) and each ACE2 ortholog are shown. Raw and fitted curves are represented by dashes and lines, respectively. The binding affinity of prototype, Delta and Omicron RBDs with ACE2 orthologs were presented as a heatmap according to the indicated color. **b** Entry of the pseudovirus of prototype and Omicron BA.1. Red fluorescence indicates BHK-21 pseudovirus-transducing cells. Untransfected BHK-21 cells were used as negative controls. Scale bar, 0.25 mm. **c** Statistics for the transduction of the prototype and Omicron BA.1 pseudoviruses. Data represent the results of 12 replicates.

To better understand the interactions between ACE2 orthologs and SARS-COV-2 variant RBDs, we assessed their binding affinities using SPR. Consistent with the flow cytometry results, Delta RBD displayed similar binding affinities to prototype RBD (Fig. 2a and Table 1). The Omicron BA.1 RBD showed a high binding affinity to ACE2 from rodents (mouse and rat), palm-civet and least horseshoe bats, as well as a lower but measurable binding affinity to lesser hedgehog tenrec. In contrast, neither the prototype nor Delta RBD showed a binding capacity to ACE2 from these species. Additionally, the Omicron BA.1 RBD displayed a slightly higher binding affinity to mouse ACE2 (mACE2) than hACE2 (14.23 ± 6.22 nM *vs*.18.2 ± 1.33 nM). The binding of the Omicron BA.1 RBD to rat, horse, big-eared horseshoe bat, fulvous fruit bat, greater horseshoe bat, little brown bat and lesser hedgehog tenrec ACE2s were positive, although lower than those detected by flow cytometry (Figs. 1, 2a).

**Table 1:** Binding affinities of the prototype, Delta, and Omicron RBDs with ACE2 orthologs from different species.

Species	Prototype (nM)	Delta (nM)	Omicron (nM)
Human	16.1 ± 1.46	21.5 ± 1.49	18.2 ± 1.33
Monkey	22 ± 4.16	16.53 ± 0.17	8.32 ± 0.53
Rabbit	48.13 ± 2.09	58.27 ± 3.40	27.27 ± 4.11
Mouse	–	–	14.23 ± 6.22
Rat	–	–	2993.33 ± 941.71
Guinea pig	–	–	–
Malayan pangolin	35.17 ± 6.53	58.2 ± 1.04	5026.67 ± 2284.39
Cat	99.05 ± 5.95	53.35 ± 7.05	24.2 ± 1.13
Civet	–	–	1147.33 ± 134.86
Fox	56.2 ± 1.53	35.65 ± 5.75	35.5 ± 6.71
Dog	100.63 ± 4.50	51.43 ± 3.90	491 ± 177.81
Raccoon dog	80 ± 5.11	124 ± 9.20	109.70 ± 20.00
Mink	305 ± 16.08	288 ± 5.12	11.88 ± 2.59
Horse	220.67 ± 20.80	78.97 ± 4.83	5893.33 ± 1208.87
Pig	138.67 ± 13.30	64.37 ± 5.70	321.33 ± 39.42
Wild Bactrian camel	103.5 ± 16.95	233 ± 20.40	38.23 ± 13.25
Alpaca	11798.53 ± 1224.84	381.35 ± 14.83	26.05 ± 2.07
Bovine	181 ± 13	71.35 ± 5.85	39.5 ± 6.94
Goat	213.5 ± 6.5	66.25 ± 0.35	126.47 ± 34.14
Sheep	174 ± 1	60.95 ± 2.35	75.13 ± 11.38
Least horseshoe bat	–	–	189.5 ± 24.5
Big-eared horseshoe bat	677.1 ± 83.87	49.56 ± 2.91	1840.22 ± 425.90
Fulvous fruit bat	156.67 ± 34.31	200.33 ± 31.85	>37700
Greater horseshoe Bat	–	–	12078 ± 677.43
Chinese horseshoe bat	–	–	–
Little brown bat	129.67 ± 23.70	185.67 ± 22.95	>55366.67
Lesser hedgehog tenrec	–	–	>29070

“–” means the binding affinity of the RBD to the ACE2 ortholog was too weak to be detected.

With evidence of binding between mouse, rat, palm-civet and least horseshoe bat ACE2s and the RBD of Omicron BA.1, which was not observed by the prototype RBD (or with lower binding affinity), we tested the entry efficiency of prototype or Omicron BA.1 pseudotyped SARS-CoV-2 engaged by these ACE2s. Consistent with the flow cytometry and SPR assays, mouse, rat and least horseshoe bat ACE2s were found to initiate the entry of Omicron BA.1 pseudovirus, but not prototype pseudovirus (Fig. 2b, c). Furthermore, cvACE2 also efficiently mediated Omicron BA.1 pseudovirus entry, but weakly mediated the prototype SARS-CoV-2 pseudovirus entry (Fig. 2b, c).

### Architecture of the Omicron BA.1 RBD complexed with mACE2 or cvACE2

The S protein of Omicron BA.1 was found to comprise 30 AA substitutions (15 on the RBD), six AA deletions and three AA insertions (Supplementary Fig. S3a). Previous work suggests that the mouse is a potential original host of Omicron BA.1^26^. In addition, neither the prototype RBD nor Delta RBD interacts with mACE2, but Omicron BA.1 RBD binds to mACE2 (14.23 ± 6.22 nM) with a higher binding affinity than that of hACE2 (18.2 ± 1.33 nM). In addition, cvACE2 also acquired a high-affinity binding capacity with Omicron BA.1 RBD (1.1 ± 0.13 μM). To evaluate the molecular basis of the broad receptor binding spectra of Omicron BA.1 RBD, we determined the cryo-EM structure of mACE2 complexed with the Omicron BA.1S protein (Supplementary Fig. S2), and the crystal structure of cvACE2 in complex with Omicron BA.1 RBD. The overall resolution of the Omicron BA.1S/mACE2 complex was 2.66 Å (Supplementary Fig. S3b and Table S1). To further improve the map quality of the Omicron BA.1 RBD/mACE2 binding interface, a mask was created to include ACE2 and RBD for local refinement and global B-factor sharpening, yielding a 3.03 Å cryo-EM map for the Omicron BA.1 RBD/mACE2 complex structure (Supplementary Fig. S3c and Table S1). The resolution of Omicron BA.1 RBD/cvACE2 complex was 3.3 Å (Supplementary Fig. S3d and Table S2).

From the complex structure, only one RBD was observed in the open conformation binding to mACE2, while the other two RBDs in the S protein were closed (Supplementary Fig. S3b). Numerous mutations on the surface of the S protein were observed, which may enable Omicron BA.1 to escape immune surveillance obtained from natural infection or vaccination (Supplementary Fig. S4a, b).

**Fig. 3:**
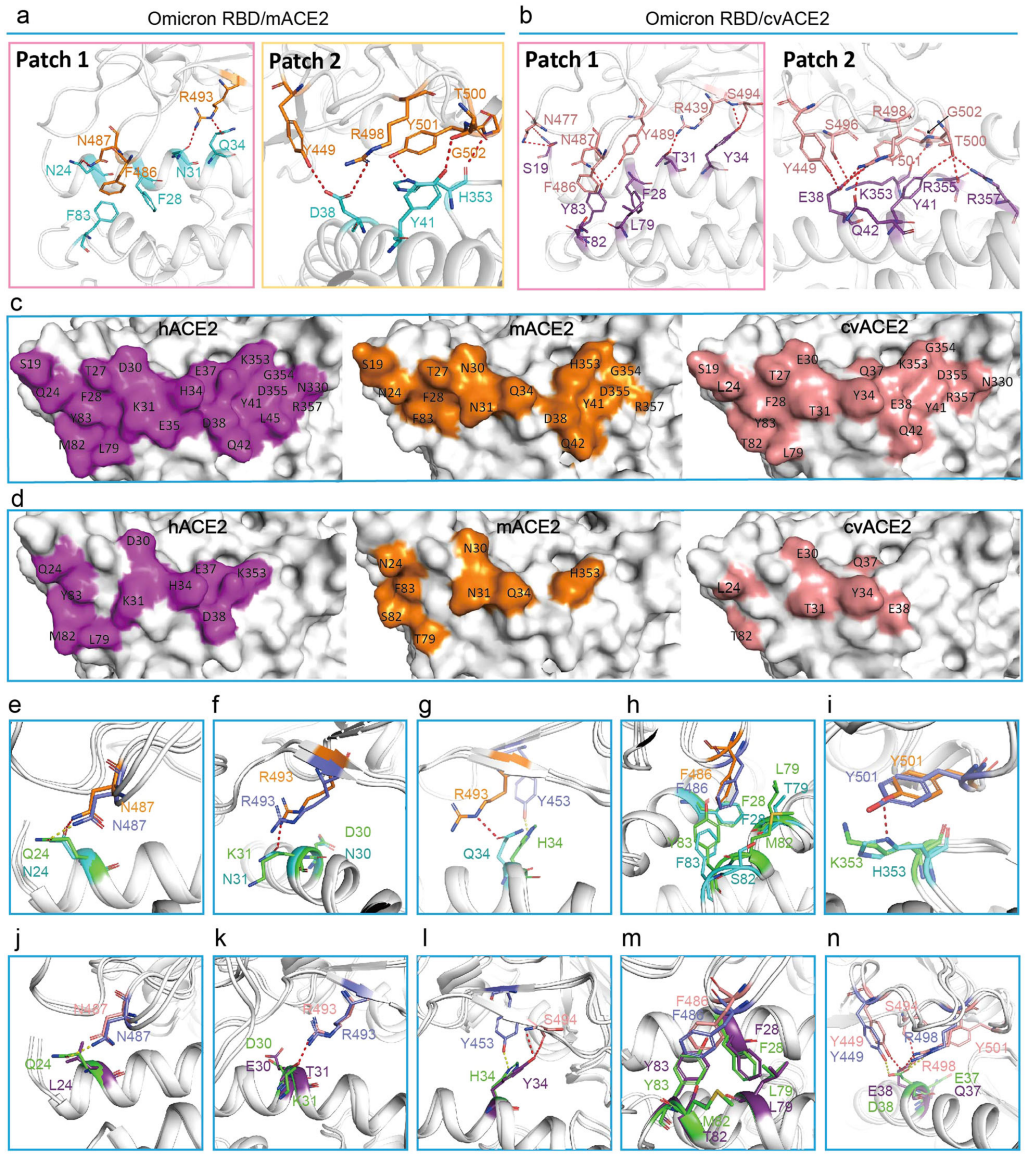
Structural basis of binding between the Omicron S protein and mouse ACE2. **a**, **b** The interacting residues of Patch 1 and 2 of Omicron BA.1 RBD/mACE2 (**a**) and Omicron BA.1 RBD/cvACE2 (**b**) are represented by sticks. H-bonds are indicated by red dashes. **c** The binding surface of hACE2 (purple, left), mACE2 (orange, middle) and cvACE2 (pink, right). **d** The eight distinct residues observed on the Omicron BA.1 RBD binding interface of ACE2 orthologs. **e**–**i** Structural comparison of the binding between Omicron BA.1 RBD and the hACE2 or mACE2. Distinct residues in the Omicron BA.1 RBD (blue)/hACE2 (green) complex, and Omicron BA.1 RBD (orange)/mACE2 (cyan) complex. Key residues are represented by sticks with the corresponding colors, and the backbone in white. H-bonds are represented by red dashes in Omicron BA.1 RBD/mACE2 and yellow dashes in Omicron BA.1 RBD/hACE2. **j**–**n** Structural comparison of the binding between Omicron BA.1 RBD and the hACE2 or cvACE2. Distinct residues in the hACE2 (green) and Omicron BA.1 RBD (salmon) complex, and cvACE2 (purple) and Omicron BA.1 RBD (light pink) complex. Key residues are represented by sticks with corresponding colors and the backbone in white. H-bonds are represented by red dashes in Omicron BA.1 RBD/cvACE2 and yellow dashes in Omicron BA.1 RBD/hACE2.

The architecture of the Omicron BA.1 RBD complexed with mACE2 resembled that of the prototype RBD/hACE2 complex but was more divergent from Omicron BA.1 RBD complex/cvACE2, with a root mean square deviation (RMSD) of 1.167 Å for 738 atoms and 2.363 Å for 787 atoms, respectively. The interacting residues of Omicron BA.1 RBD with both mACE2 and cvACE2 could be divided into two patches (Patch 1 and 2) (Fig. 3a, b), similar to the prototype RBD binding to hACE2^17^. In Patch 1 of the Omicron BA.1 RBD/mACE2 complex, R493 of Omicron BA.1 RBD formed hydrogen bonds (H-bonds) with N31 and Q34 of mACE2. N487 of Omicron BA.1 RBD contacted N24 of mACE2 (Fig. 3a and Table 2). In addition, F486 formed a π-π stack with both F28 and F83 of mACE2 (Fig. 3a and Table 2). Patch 2 included an H-bond network formed by Y449, R498, Y501, and G502 of the Omicron BA.1 RBD and D38, Y41, and H353 of mACE2 (Fig. 3a and Table 2).

**Table 2:** Amino acid residue comparison of Omicron RBD and MASCp36 RBD interacting with mACE2.

mACE2	Omicron-RBD	MASCp36-RBD
S19 (1/0)	A475 (1)	–
N24 (16/6)	A475 (4), G476 (2), N487 (10, **1**)	A475 (2), G476 (2), N487 (2)
T27 (9/8)	F456 (4), Y473 (1), Y489 (4)	F456 (3), Y473 (1), Y489 (4)
F28 (6/9)	F486 (2), Y489 (4)	Y489 (9)
N30 (2/3)	F456 (2),	L455 (1), F456 (2)
N31 (20/17)	F456 (3), Y489 (11), R493 (6, **1**)	F456 (4), Y489 (8), H493 (5)
Q34 (24/13)	Y453 (2), L455 (2), R493 (19, **1**), S494 (1)	N417 (3), Y453 (6, **1**), L455 (3), H493 (1)
E35 (0/12)	–	H493 (12, **1**)
D38 (12/4)	Y449 (5, **1**), S496 (3), R498 (3, **1**), Y501 (1)	S494 (1), Y495 (1), G496 (1), Y501 (1)
Y41 (23/31)	R498 (7), T500 (6, **1**), Y501 (10)	Q498 (3), T500 (11, **1**), Y501 (17)
Q42 (4/4)	Y449 (1), R498 (3)	Q498 (4, **1**)
L45 (0/3)	–	Q498 (1), T500 (2)
T79 (0/2)	–	F486 (2)
S82 (0/5)	–	F486 (4), N487 (1)
F83 (5/0)	F486 (5)	–
T324 (0/1)	–	V503 (1)
N330 (0/4)	–	T500 (4)
H353 (48/50)	Y501 (26, **1**), G502 (4, **1**), H505 (18)	Y501 (18), G502 (3), Y505 (29)
G354 (7/9)	G502 (6), H505 (1)	Y501 (3), G502 (6)
D355 (5/10)	T500 (5)	T500 (10, **2**)
R357 (2/7)	T500 (2)	T500 (7, **1**)
R393 (0/1)	–	Y505 (1)
Total	184, **8**	199, **7**

The numbers in parentheses of Omicron RBD and MASCp36-RBD residues represent the number of Van der Waals contacts between the indicated residues with hACE2. The numbers with underline suggest numbers of potential H-bonds between the pairs of residues. Van der Waals contact was analyzed at a cutoff of 4.5 Å, H-bonds and salt bridges at a cutoff of 3.5 Å. “–” represents that these amino acids in the mACE2 do not interact with the corresponding RBD.

In the Omicron BA.1 RBD/cvACE2 complex, Patch 1 was found to involve an H-bond network between S19, T31, Y34, and Y83 of cvACE2 and R439, N477, N487, Y489, and S494, as well as π-π stacking interactions among F486 of Omicron BA.1 RBD and F28 and Y83 of cvACE2 (Fig. 3b and Table 3). In Patch 2, the H-bond network between E38, Y41, Q42, R355, and R357 of cvACE2 and Y449, S496, R498, T500, and Y501 of Omicron BA.1 RBD was observed (Fig. 3b and Table 3).

**Table 3:** Amino acid residue comparison of Omicron RBD interacting with cACE2.

civet ACE2	Omicron-RBD
S19	A475 (3), G476 (5), N477 (13, **2**)
L24	A475 (4), G476 (2), N487 (13), Y489 (1)
T27	F456 (6), Y473 (2), A475 (3), Y489 (8)
F28	Y489 (9)
E30	L455 (4), F456 (8)
T31	F456 (2), Y489 (8), R493 (5, **1**)
Y34	Y453 (2), R493 (22), S494 (15, **1**), S496 (1)
Q37	H505 (1)
E38	Y449 (7, **1**), S496 (9, **1**), R498 (9, **5**), Y501 (3, **1**)
Y41	R498 (3), R498 (1), T500 (9, **2**), Y501 (17)
Q42	Y449 (2, **1**), R498 (5, **1**)
L79	F486 (2)
T82	F486 (4)
Y83	F486 (11), N487 (7, **1**), Y489 (2, **1**)
N330	T500 (8, **1**)
K353	S496 (1), Y501 (26), G502 (6, **1**), H505 (25)
G354	G502 (8), H505 (4)
D355	T500 (12), G502 (3)
R357	T500 (5, **1**)
Total	326, **21**

The numbers in parentheses of Omicron RBD residues represent the number of Van der Waals contacts between the indicated residues with cACE2. The numbers with underline suggest numbers of potential H-bonds salt bridges between the pairs of residues. Van der Waals contact was analyzed at a cutoff of 4.5 Å, H-bonds and salt bridges at a cutoff of 3.5 Å.

We further compared the interface residues of mACE2 and cvACE2 with hACE2 binding to Omicron BA.1 RBD (Fig. 3c)^20^. Compared with hACE2, eight substitutions were observed at the mACE2 interface: Q24 (human)/N24 (mouse), D30/N30, K31/N31, H34/Q34, L79/T79, M82/S82, Y83/F83 and K353/H353 (Fig. 3d; Supplementary Fig. S4c). Structural analysis showed no significant changes in the molecular contacts of N24/Q24 and N30/D30 (Fig. 3e; Supplementary Fig. S4c), whereas N31 of mACE2 was found to form an H-bond with R493; however, a repulsive interaction existed between K31 of hACE2 and R493 (Fig. 3f). In contrast, H34 of hACE2 formed an H-bond with Y453 of Omicron BA.1 RBD, whereas Q34 of mACE2 formed an H-bond with R493 (Fig. 3g; Supplementary Fig. S4c). Both F83 of mACE2 and Y83 of hACE2 were found to be involved in hydrophobic patch formation. However, T79 and S82 of mACE2 were not implicated, while L79 and M82 of hACE2 were (Fig. 3h). An additional H-bond was observed between H353 of mACE2 and Y501 of the Omicron BA.1 RBD, which was absent between K353 of hACE2 and Y501 (Fig. 3i). H353 was only found to exist in mouse and rat ACE2s among the 27 ACE2 orthologs (Supplementary Fig. S5).

For cvACE2, seven substitutions were observed: Q24 (human)/L24 (palm-civet), D30/E30, K31/T31, H34/Y34, E37/Q37, D38/E38 and M82/T82 (Fig. 3c, d). Q24 of hACE2 formed an H-bond with N487 of the RBD, whereas L24 of cvACE2 did not (Fig. 3j). No significant change was observed between D30 of cvACE2 and E30 of cvACE2, whereas T31 of cvACE2 formed an H-bond with R493 (Fig. 3k). H34 of hACE2 formed an H-bond with Y453 of the RBD, whereas Y34 of cvACE2 formed two H-bonds with S494 of the RBD (Fig. 3l). In contrast, the hydrophilic T82 of cvACE2 may sabotage the hydrophobic patch comprising F28, L79, and Y83 of cvACE2 and F486 of the RBD, whereas M82 of hACE2 may participate in the hydrophobic patch (Fig. 3m). D38/E38 displayed a different H-bond network, where D38 of hACE2 interacts with Y449 and R498, whereas E38 of cvACE2 formed H-bonds with Y449, S494, R498, and Y501 of the RBD (Fig. 3n).

### Effect of key Omicron BA.1 RBD residues on host range expansion

**Fig. 4:**
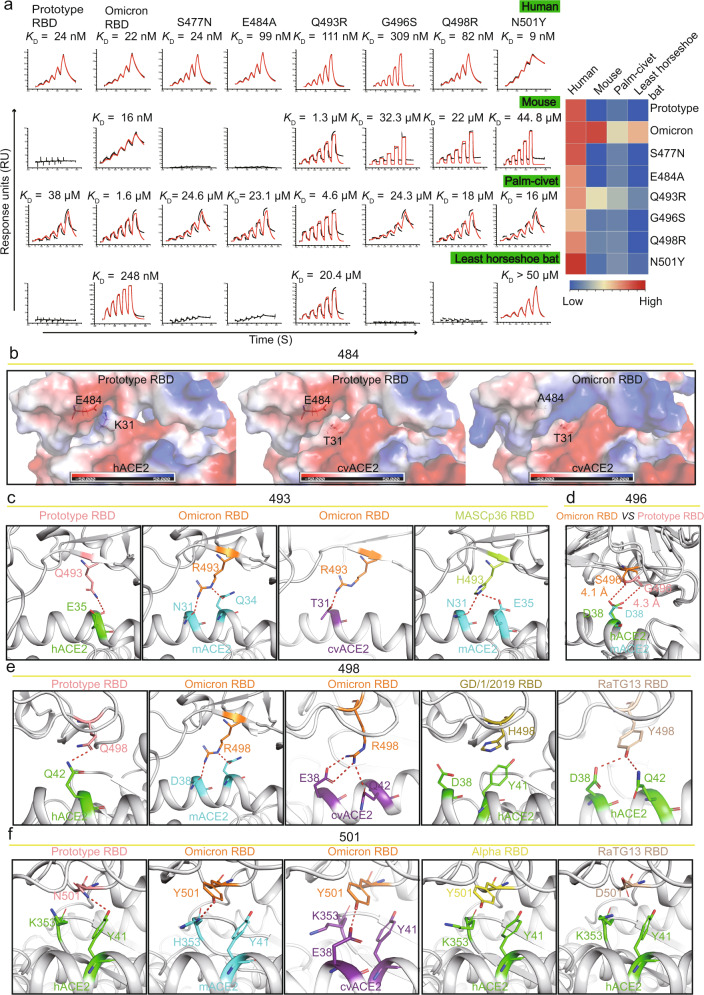
Mutational and structural analysis of key residues responsible for Omicron BA.1 RBD binding. **a** SPR analysis of binding between the six prototype RBD mutants and ACE2s from human, mouse, palm-civet and least horseshoe bat. Prototype SARS-CoV2 RBD and Omicron BA.1 RBD were used as the controls. Raw and fitted curves are represented by black and red lines. The binding affinity of prototype, Omicron and prototype mutant RBDs to human, mouse, palm-civet and least horseshoe bat ACE2s were presented as a heatmap according to the indicated color. **b** The surface of prototype RBD/hACE2 (left), prototype RBD/hACE2 (middle) and Omicron BA.1 RBD/cvACE2 (right) are colored for electrostatic potential: blue (basic), white (neutral) and red (acidic). Residues located on site 484 of the RBD and site 31 of the ACE2 are represented by sticks. **c**–**f** Structural details of residue 493 (**c**), 496 (**d**), 498 (**e**) and 501 (**f**). The interacting residues of hACE2 and mACE2 are colored in green and cyan, respectively. The prototype RBD, Omicron BA.1 RBD, MASCp36 RBD, GD/1/2019 RBD, Alpha RBD, and RaTG13 RBD are colored in salmon, orange, pink, pink, yellow, and wheat, respectively. The H-bonds are represented by red dashes.

To determine why Omicron BA.1 RBD displays an expanded host range, we constructed nine mutants of the prototype RBD containing a single-site substitution on the hACE2-recognizing interface, namely K417N, G446S, S477N, E484A, Q493R, G496S, Q498R, N501Y, and Y505H. The wild-type and mutated prototype RBD, as well as the Omicron BA.1 RBD protein, were purified and their affinities to ACE2 orthologs from human, mouse, palm-civet, and least horseshoe bat were measured. Corresponding to a previous work^20^, N501Y, which is the only mutation site in the Alpha SARS-CoV-2 variant RBD, was found to increase its binding affinity to hACE2 by 2.7-fold. However, the K417N, G446S, E484A, Q493R, G496S, Q498R, and Y505H mutants demonstrated significantly decreased binding affinities for hACE2 (Fig. 4a; Supplementary Fig. S6). The S477N mutant showed similar binding affinities to Omicron BA.1 RBD (Fig. 4a). Q493R, G496S, Q498R, or N501Y substitutions in the prototype RBD independently confer binding capacity to mACE2. Notably, a synergetic effect was observed for the four substitutions, where individual substitution only resulted in weak binding (1.3 μM–44.8 μM), but which enabled strong binding when coordinated, with a *K*_D_ of 16 nM (Fig. 4a). E484A, Q493R, and N501Y could facilitate cvACE2 binding (Fig. 4a). Q493R and N501Y may independently enable the prototype RBD to bind to ACE2 from the least horseshoe bat, and a similar synergetic effect was observed (Fig. 4a).

To elucidate the molecular mechanism underlying the binding capacity of Omicron BA.1 RBD for mACE2, we compared the molecular contacts among Omicron BA.1 RBD/cvACE2, Omicron BA.1 RBD/mACE2 and prototype RBD/hACE2 complexes. Although E484A is located outside the binding interface between the RBD and ACE2, surface charge alteration may still have an impact on binding affinity. When hACE2 binds to the prototype RBD, the charge attraction between E484 of RBD and K31 of hACE2 facilitates the interaction. However, when the prototype RBD or omicron RBD binds to cvACE2, the small side chain of T31 could neither bind to E484 nor A484 in the RBD (Fig. 4b). Regarding mACE2, it is worth noting that three of the four substitutions enabling the Omicron BA.1 RBD to bind to mACE2 were included in Patch 2 (Supplementary Fig. S4c). We then analyzed the structural details of these four enabling substitutions. Instead of forming an H-bond with E35, similar to Q493 of the prototype RBD, R493 of the Omicron BA.1 RBD formed H-bonds with N31 and Q34, respectively (Fig. 4c). Notably, the S protein of a lethal mouse-adapted SARS-CoV-2 strain, MASCp36, also carried a Q493H substitution, which emerged after N501Y during in vivo passage in mice, increasing the binding affinity to mACE2 by 27.8-fold (*K*_D_ of 353.4 μM vs 12.67 μM)^33^. A structural analysis of MASCp36 H493 revealed an H-bond with N31 and a salt bridge with E35 of mACE2 (Fig. 4c). Although neither G496 of the prototype RBD nor S496 formed H-bonds with mACE2, the relatively longer side chain of S496 shortened its distance to D38 of mACE2 and formed additional van der Waals force contacts compared to G496 of the prototype RBD (Fig. 4d and Table 2). Moreover, the longer side chain of R498 of Omicron BA.1 RBD formed an additional salt bridge with D38 of mACE2. Similarly, R498 also promoted RBD binding to cvACE2, where the longer side chain of cvACE2 E38 formed an additional H-bond with R498 (Fig. 4e). R498 was also found to result in a marked increase in the positive charge on the RBD surface, which enhanced the binding affinity between Omicron BA.1 RBD and mACE2 (Supplementary Fig. S6b). A similar effect was previously described in a study on GD/1/2019 RBD (Fig. 4e)^34^. Site 501 was identified as a mutation hotspot and host range determinant^5^. As previously described^28^, the N501Y substitution endowed it with many favorable noncovalent interactions, such as a cation-π interaction with hACE2 K353 and a π-π stacking interaction with hACE2 Y41. H353 of mACE2, in contrast, resulted in even stronger binding as a result of the formation of an additional H-bond with Y501 of the Omicron BA.1 RBD (Fig. 4f). In the Omicron BA.1 RBD/cvACE2 complex, Y501 of the RBD formed an H-bond with E38, which strengthened the RBD interaction (Fig. 4f). It is also worth noting that the RBD of RaTG13, one of the closest relatives of SARS-CoV-2, carries D501, which preferentially binds H353 to K353 (Fig. 4f)^19^.

## Discussion

In both natural and experimental settings, cases of SARS-CoV-2 transmission from humans to other mammals have been reported^5^. The interspecies transmission of SASR-CoV-2 promotes virus evolution and poses a severe threat to public health^5^. In this study, the receptor recognition of Omicron BA.1 RBD to 27 ACE2 orthologs, including hACE2, was evaluated. As a result, compared to the prototype and Delta RBDs, Omicron BA.1 RBD was found to expand its receptor-binding spectra to palm-civet, mouse, rat, more bats (least horseshoe bat and greater horseshoe bat) and lesser hedgehog tenrec ACE2s.

As reported in our previous work^20^, the binding affinity of Omicron BA.1 RBD with hACE2 was found to be similar to that of the prototype RBD. Herein, we evaluated the binding affinity of each single-site substitution on the binding surface of RBD to hACE2 and found that seven out of nine substitutions (K417N, G446S, E484A, Q493R, G496S, Q498R, and Y505H) reduced the binding capacity of Omicron BA.1 RBD to hACE2, while one substitution (S477N) had no significant effect on the binding affinity. Only the N501Y substitution increased the binding affinity of the Omicron RBD to hACE2. Combining these nine substitutions, Omicron BA.1 RBD showed a binding affinity comparable to that of the prototype for hACE2. Genome analysis demonstrated that the Omicron BA.1 RBD contained multiple substitutions responsible for immune escape^18,35^. Multiple studies have reported the emergence of Q493R during treatment with FDA-approved bamlanivimab/etesevimab^31,36^. Consistently, it has also been reported that the Omicron BA.1 strain demonstrates extensive escape of neutralizing antibodies and sera from convalescent patients or vaccinated people^37–39^, which indicates that the extensive documented mutations in Omicron may arise from the coordinated evolution of immune escape and receptor binding.

The Q493K, Q498H, and N501Y substitutions in the SARS-CoV-2 RBD have been identified in mouse-adapted strains^40–42^. Notably, Q498R was observed during the in vitro evolution of the RBD and was found to be epistatic to N501Y, with the two substitutions together increasing the ACE2-binding affinity by ~600-fold^29^. Herein, we found that the Q498R substitution changed the charge of Patch 2 in the Omicron RBD and enhanced its binding affinity to mACE2. Furthermore, we found that the S477N, E484A, and G496S substitutions also play a vital role in expanding the receptor-binding spectra of the Omicron BA.1 RBD.

The N501Y mutation is a hotspot that has been reported in Alpha, Beta, and Gamma RBDs, enhancing the binding of the SARS-CoV-2 RBD to hACE2 by forming a π-π stacking interaction with Y41 of hACE2^28^. Herein, we found that Q493R and N501Y promoted contact between Omicron BA.1 RBD with the ACE2s of mouse, palm-civet, and least horseshoe bat. While K353 of ACE2 is a conserved residue among the 27 ACE2 orthologs tested, K353 is substituted by H353 in mouse and rat ACE2s, which enhances the binding of mACE2 to the Omicron BA.1 RBD via an additional H-bond with Y501 of the RBD. In addition, the G496S mutation was also found to increase the binding of RBD to mACE2, but not palm-civet and least horseshoe bat ACE2s.

Palm-civet is reported to infect SARS-CoV during SARS-CoV outbreak in 2002–2003, but the binding affinity of palm-civet ACE2 with SARS-CoV-2 RBD is hardly detected and palm-civet ACE2 mediates weakly entry of pseudotyped prototype SARS-CoV-2^13,43^. Herein, both SPR and VSV-based pseudovirus infection assays confirmed that Omicron BA.1 RBD poses a great risk to the palm-civet. In addition, E484A, Q493R, Q498R, and N501Y were found to synergistically enhance the binding affinity of Omicron BA.1 RBD to cvACE2 and broaden the host range of SARS-CoV-2. Compared to hACE2, seven substitutions were observed on the binding surface of cvACE2. The interaction network of Omicron BA.1 RBD with cvACE2 was rearranged to enhance its binding affinity to cvACE2.

The mouse is also regarded as a potential host of the Omicron variant, and it has been hypothesized that the Omicron variant is a mouse-adapted virus^24^. The rapid replication and close-contact transmission of the Omicron virus in mice have been experimentally observed, resulting in severe lung lesions and inflammatory responses^27^. Herein, the binding affinity of Omicron BA.1 RBD to mACE2 was found to be significantly increased (~2800-fold) compared to the mouse-adapted N501Y mutant variant^26^. One possible explanation for the origin of SARS-CoV-2 is that a mouse-adapted SARS-CoV-2 variant, such as Alpha, Beta, and Gamma variants, or other variants that are yet undetected, were transmitted to mice from humans, thereafter evolving into Omicron in mice. Subsequently, at the right time, the virus was re-transmitted back to humans. Notably, two key mouse-adapted mutations, Q493R and Q498R, had not been previously reported in any other SARS-CoV-2 variant before the discovery of Omicron, suggesting that mice are potentially the original host of Omicron.

Natural SARS-CoV-2 infections have been detected in several animals, the majority of which have been domestic or zoo animals. While these animals were relatively easy to test and monitor, the infection of wild animals with SARS-CoV-2 poses a much greater challenge to the control of infection. For example, a rate of SARS-CoV-2 infection of up to 70% has been reported in wild white-tailed deer in North America and the Omicron variant has yet been detected in wild white-tailed deer^8,44^. Its broad potential host range highlights the high risk of transmissivity to wild animals, including mice and bats. Therefore, continuously surveying Omicron variants in potential animal reservoirs is crucial to prevent interspecies transmission.

Although receptor binding plays a pivotal role in SARS-CoV-2 infection, other residues in the S protein may also influence viral entry. For example, D614G enhances the infection of SARS-CoV-2 by stabilizing the S protein^45^. Therefore, other mutations in the S protein of Omicron BA.1 may also influence its entry. In addition, other co-receptors and co-factors may also play a role in Omicron BA.1 infection, such as transmembrane protease serine 2 (TMPRSS2) in the cell surface pathway and sorting nexin 27 (SNX27) in the endocytic pathway^46^, which will need to be addressed in future studies.

In sum, our work here indicates that the omicron variant has the potential new animal hosts and RBD-receptor complex structures defined the molecular basis for expanding host receptor binding.

## Materials and methods

### Gene cloning

The full-length coding sequences of 27 ACE2 orthologs were subcloned into the pEGFP-N1 vector for flow cytometry assays. For protein purification, the extracellular domains of these ACE2 orthologs (residues 1–740) fused with the Fc domain of mouse IgG (mFc) were subcloned into the pCAGGS vector, as previously reported^13^. The peptidase domains of several ACE2 orthologs (residues 19–615) with a His-tag were inserted into the *NdeI* and *XhoI* sites of pET-21a (+) or were fused to a signal peptide of IL10 and then inserted into the *EcoRI* and *XhoI* sites of pCAGGS. These ACE2 orthologs were from human, monkey, rabbit, guinea pig, mouse, rat, Malayan pangolin, cat, palm-civet, fox, dog, raccoon dog, horse, pig, Bactrian camel, alpaca, bovine, goat, sheep, little brown bat, fulvous fruit bat, greater horseshoe bat, Chinese horseshoe bat, least horseshoe bat, big-eared horseshoe bat and lesser hedgehog tenrec and mink.

The Omicron BA.1S protein sequence (residues 1–1205) was fused with a C-terminal foldon tag, followed by a Strep-II tag and a His-tag. The “6P”-mutations (F814P, A889P, A896P, A939P, K983P, and V984P) were introduced to stabilize the profusion state^47^. After codon optimization, the gene sequence was subcloned into the pCAGGS vector.

The coding sequences of the SARS-CoV-2 prototype RBD (residues 319–541, GISAID: EPI_ISL_402119), Delta RBD (residues 319–541, EPI_ISL_2020954) and Omicron BA.1 RBD (residues 319–541, EPI_ISL_6640916) were subcloned into the pCAGGS vector with an N-terminal signal peptide (residues 1–15 of the SARS-CoV-2 S protein) and a C-terminal His-tag. A series of subclones containing single-point mutations K417N, G446S, S477N, E484A, Q493R, G496S, Q498R, N501Y, and Y505H were synthesized using GenScript based on the prototype RBD plasmid.

The full-length coding sequences of SARS-CoV-2 prototype S (GISAID: EPI_ISL_402119) and Omicron BA.1S (EPI_ISL_6640916) were cloned into the pCAGGS vector for pseudovirus preparation.

All the genes used in this study are listed in Supplementary Table S3.

### Protein expression and purification

Plasmids containing ACE2s with an mFc-tag were transiently transfected into HEK293F cells. After five days, the ACE2s were captured from the supernatants using HiTrap^TM^ Protein A HP (GE Healthcare) chromatography and eluted with 100 mM glycine (pH 3.0). ACE2s and His-tagged RBDs were transiently transfected into HEK293F cells. After five days, the supernatants containing proteins were collected, filtered, and purified using HisTrap^TM^ Excel columns (GE Healthcare). All proteins obtained by affinity chromatography were further purified by gel filtration using a HiLoad 16/600 Superdex^TM^ 200 pg column and ÄKTA System (GE Healthcare). Purified proteins were stored in PBS buffer (1.8 mM KH_2_PO_4_, 10 mM Na_2_HPO_4_ (pH 7.4), 137 mM NaCl and 2.7 mM KCl). The expression and purification procedure of the Omicron BA.1S protein was similar to that of RBDs. However, the expression period was reduced to three days, and a Superose 6 Increase 10/300 GL column (GE Healthcare) was used for size exclusion chromatography (SEC).

The mACE2 (residues 19‒615) plasmid was transformed into competent *E. coli* strain BL21 (DE3) cells. The positive monoclonal bacteria were picked and incubated at 37 °C, followed by the addition of Isopropyl β-D-Thiogalactoside (IPTG; Sigma-Aldrich) to the medium during the logarithmic growth period (OD_600_: 0.4-0.6). After centrifugation and ultrasonic treatment, inclusion bodies were dissolved in 6 M guanidine hydrochloride and slowly dripped into a refolding buffer (100 mM Tris, 400 mM L-arginine, 2 mM EDTA, pH 8.0) overnight. After purification by gel filtration using a HiLoad 16/600 Superdex^TM^ 200 pg column, mACE2 protein was stored in protein buffer (20 mM Tris-HCl, 150 mM NaCl, pH 8.0).

Purified S protein and mACE2 were mixed at a 1:5 molar ratio and incubated on ice for 2 h before SEC using a Superose 6 Increase 10/300 GL column (GE Healthcare). The central fraction of the compound peak was collected and analyzed by SDS-PAGE.

### Flow cytometry assays

Plasmids containing ACE2 orthologs and pEGFP-N1 vectors were transiently transfected into baby hamster kidney (BHK-21) cells. The cells were collected 24 h after transfection, resuspended in PBS, and incubated with 10 mg/mL of test proteins (prototype SARS-CoV-2 RBD, Delta RBD and Omicron BA.1 RBD) at room temperature for 30 min. Subsequently, the cells were washed thrice with PBS and incubated with anti-His/APC antibody (1:500 dilution; Miltenyi Biotec) at room temperature for 30 min. Finally, the cells were washed thrice with PBS and analyzed by flow cytometry assay using BD FACS Calibur flow cytometer (BD Biosciences). Figures were generated and analyzed using FlowJo 10.6 (TreeStar Inc., Ashland, OR, USA).

### SPR analysis

To determine the affinity between ACE2 orthologs and RBDs, ACE2-mFc proteins were immobilized on CM5 sensors (GE Healthcare). As the flow phases, the prototype, Delta and Omicron BA.1 RBDs were doubly diluted into five stages of concentration and then interacted with the CM5 sensor using a single cycle mode generated by the BIAcore 8 K control system (GE Healthcare).

Human, mouse, palm-civet, and least horseshoe bat were chosen as the typical species. To compare the binding differences between prototype RBD mutations and these species, the RBDs were immobilized on CM5 sensors (GE Healthcare), and the peptidase domains of the four ACE2s were serially diluted as the flow phases, using the same mode as mentioned above.

For all measurements, PBST (PBS, pH 7.4, 0.5‰ (v/v) Tween-20) was used as the running buffer. Kinetics or steady states were analyzed using the BIAcore™ Insight software (GE Healthcare) using a 1:1 binding model. The appropriate immobilization levels and concentrations of the solutions were set (Supplementary Tables S4 and S5). Graphics were generated using OriginPro 9.1.

### Production and quantification of pseudoviruses

The SARS-CoV-2 prototype and Omicron BA.1 pseudoviruses were constructed with a mCherry-encoding replication-deficient vesicular stomatitis virus (VSV) vector backbone (VSV-ΔG-mCherry). HEK293T cells were transfected with 30 μg of the plasmid for S protein expression. VSV-ΔG-mCherry pseudoviruses were added 24 h after transfection. The inoculum was removed after incubating for 1 h at 37 °C. The culture medium was then changed to DMEM supplemented with 10% FBS and 10 μg/mL anti-VSV-G antibody (I1‐Hybridoma ATCC^®^ CRL2700) after washing the cells with PBS. The pseudoviruses were harvested 30 h after inoculation, filtered, aliquoted and stored at −80 °C.

### Pseudovirus infection assay

pEGFP-N1 vectors containing the *ACE2* gene of human, mouse, rat, palm-civet, or least horseshoe bats were transfected into BHK-21 cells. After 24 h, eGFP-positive cells were sorted and seeded in 96-well plates at 2 × 10^4^ cells per well using a BD FACS Aria III Flow Cytometer (BD Biosciences) and then cultivated for another 24 h before pseudovirus infection.

The pseudovirus particles of the SARS-CoV-2 prototype and Omicron BA.1 were normalized to the same amount for quantitation by qRT-PCR. Next, 100 μL of each pseudovirus was added to each well of a 96-well plate containing sorted cells. BHK-21 cells that were not transfected were used as controls. Fifteen hours after transfection, the plates were imaged and the number of fluorescent cells was counted using a CQ1 confocal image cytometer (Yokogawa). Each group consisted of 12 replicates.

### Cryo-EM sample preparation and data acquisition

To prepare the cryo-EM samples, 4.0 μL of the Omicron S/mACE2 complex at ~1.0 mg /mL was applied to 1.2/1.3 Au Quantifoil grids that were glow discharged for 20 s at 15 mA. The grids were immediately plunge-frozen in liquid ethane using a Vitrobot Mark IV (Thermo Fisher Scientific), and excess protein was blotted away with a blotting time of 8 s and a blotting force of −10 at 4 °C in 100% humidity. The prepared grids were transferred to a 300 kV Titan Krios transmission electron microscope equipped with a Gatan K3 detector and a GIF quantum energy filter. Movies were collected at a magnification of 81,000× with a calibrated pixel size of 1.1 Å over a defocus range of ‒1.0 μm to ‒2.0 μm in super-resolution counting mode with a total dose of 60 e^-^/Å^2^ using EPU automated acquisition software.

### Image processing and 3D reconstruction

Using MotionCor2 v1.2.4, 5812 raw movies were motion corrected^48^. The micrograph contrast transfer function (CTF) correction parameters were estimated using patch CTF estimation^49^ implemented in cryoSPARC v.3.3.1^50^. We first applied 2341 micrographs for reference-free particle picking using Blob picker in cryoSPARC, and 333,646 particles were picked and extracted for 2D classification. After 2D classification, the best class averages were used to generate the initial 3D reconstructions and further processed for heterogeneous refinement. Then reference particles from the two best volumes were used as a training dataset for the optimization of a convolutional neural network in the automated particle picking software Topaz^51^. Topaz-extracted particles (2,382,962) were selected from the full set of 5812 micrographs and subjected to three rounds of iterative 2D classification, and a clean set of 1,329,538 particles was selected to perform homogeneous refinement in cryoSPARC using the previously generated initial models. Six distinct classes (class = 6) were found, and the dominant two classes (48% and 50%) were further refined using non-uniform refinement to yield the two 2.66 Å and 2.64 Å overall cryo-EM maps, respectively.

To improve the map quality of the RBD/mACE2 binding interface, a mask was created to include ACE2 and RBD for two rounds of iterative local refinement and global B-factor sharpening, which yielded a 3.03 Å cryo-EM map for the RBD/mACE2 structure. The image-processing workflow is summarized in Supplementary Fig. S2. Details of the overall resolution and locally refined resolutions according to the gold-standard FSC can be found in Supplementary Table S1.

### Model building and structure refinement

To model the entire Omicron S/mACE2 complex, the SARS-CoV-2 S trimer with the peptidase domain (PD) of hACE2 (PDB code 7XD7) was fitted into the 2.66 Å overall cryo-EM map using UCSF Chimera v.1.15^52^. Mutations and manual adjustments were performed using the COOT v.0.9.3^53^. Glycans were added to the N-linked glycosylation sites in coot. For the RBD/ACE2 complex, the model was built using published coordinates (PDB code 6LZG) with Phenix and Coot based on the 3.03 Å focus-refined cryo-EM map, the majority of which was clearly visible in the cryo-EM map. Each residue was manually checked with the chemical properties considered during model building. Structural refinement was performed using Phenix^54^ with secondary structure and geometry restraints to prevent overfitting. Molprobity^55^ was used to validate the geometry and evaluate the structural quality. The statistics associated with data collection, 3D reconstruction, and model building are summarized in Supplementary Table S1.

### Crystallization, data collection, and structure determination

The crystallization of the Omicron RBD/cvACE2 complex was performed using the vapor-diffusion sitting-drop method, with 0.8 μL of protein mixing with 0.8 μL of reservoir solution at 18 °C. High-resolution crystals were obtained using 0.2 M potassium thiocyanate and 20% w/v polyethylene glycol 3350. Diffraction data were collected at the Shanghai Synchrotron Radiation Facility (SSRF) BL10U2. The data were indexed, integrated, and scaled using HKL2000^56^. The structure of the Omicron RBD/cvACE2 was determined by the molecular replacement method using Phaser^57^ with the previously reported structure of the SARS-CoV-2 prototype RBD/hACE2 (PDB: 6LZG) as the search model. Atomic models were built using Coot^53^ and refined using Phenix^54^. Data collection, processing, and refinement statistics are summarized in Supplementary Table S1. The structure was analyzed using PyMOL (https://pymol.org/2/).

## Supplementary information

Supplementary Information

## Data Availability

The atomic coordinates for the cryo-EM structure of the Omicron BA.1S protein/mACE2 complex (PDB code: 7WRH), the local refinement and global B-factor sharpening of Omicron RBD/mACE2 complex (PDB code: 7WRI) and the Omicron BA.1 RBD/cvACE2 complex (PDB code: 7WSK) have been deposited in the Protein Data Bank (www.rcsb.org).

## References

[1] Karim SSA, Karim QA (2021). Omicron SARS-CoV-2 variant: a new chapter in the COVID-19 pandemic. Lancet.

[2] Li J, Lai S, Gao GF, Shi W (2021). The emergence, genomic diversity and global spread of SARS-CoV-2. Nature.

[3] World Health Organization. Update on omicron. Available from: https://www.who.int/news/item/28-11-2021-update-on-omicron.

[4] Xu, Z., Liu, K. & Gao, G. F. Omicron variant of SARS-CoV-2 imposes a new challenge for the global public health. *Biosaf Health*10.1016/j.bsheal.2022.01.002 (2022). 10.1016/j.bsheal.2022.01.002 PMC8767758 35072038

[5] Gao, G. F. & Wang, L. COVID-19 expands its territories from humans to animals. *China CDC Wkly.* **3**, 855–858 (2021). 10.46234/ccdcw2021.210 PMC8521158 34703641

[6] Jo WK (2021). Potential zoonotic sources of SARS-CoV-2 infections. Transbound. Emerg. Dis..

[7] Barrs VR (2020). SARS-CoV-2 in quarantined domestic cats from COVID-19 households or close contacts, Hong Kong, China. Emerg. Infect. Dis..

[8] Chandler, J. C. et al. SARS-CoV-2 exposure in wild white-tailed deer (Odocoileus virginianus). *Proc. Natl. Acad. Sci. USA* **118**, e2114828118 (2021). 10.1073/pnas.2114828118 PMC8617405 34732584

[9] Oude Munnink BB (2021). Transmission of SARS-CoV-2 on mink farms between humans and mink and back to humans. Science.

[10] Oreshkova, N. et al. SARS-CoV-2 infection in farmed minks, the Netherlands, April and May 2020. *Euro Surveil.* **25**, 2001005 (2020). 10.2807/1560-7917.ES.2020.25.23.2001005 PMC7403642 32553059

[11] Sit THC (2020). Infection of dogs with SARS-CoV-2. Nature.

[12] Liu, Y. et al. Functional and genetic analysis of viral receptor ACE2 orthologs reveals a broad potential host range of SARS-CoV-2. *Proc. Natl. Acad. Sci. USA* **118**, e2025373118 (2021). 10.1073/pnas.2025373118 PMC8000431 33658332

[13] Wu, L. et al. Broad host range of SARS-CoV-2 and the molecular basis for SARS-CoV-2 binding to cat ACE2. *Cell Discov.* **6**, 68 (2020). 10.1038/s41421-020-00210-9 PMC7526519 33020722

[14] Yan H (2021). ACE2 receptor usage reveals variation in susceptibility to SARS-CoV and SARS-CoV-2 infection among bat species. Nat. Ecol. Evol..

[15] Genovese, L., Zaccaria, M., Farzan, M., Johnson, W. & Momeni B. Investigating the mutational landscape of the SARS-CoV-2 Omicron variant via ab initio quantum mechanical modeling. *bioRxiv*10.1101/2021.12.01.470748 (2021).

[16] Qin, S. et al. Genome characterization and potential risk assessment of the novel SARS-CoV-2 variant Omicron (B. 1.1. 529). *Zoonoses*10.15212/ZOONOSES-2021-0024 (2021).

[17] Wang, Q. et al. Structural and functional basis of SARS-CoV-2 entry by using human ACE2. *Cell* **181**, 894–904.e9 (2020). 10.1016/j.cell.2020.03.045 PMC7144619 32275855

[18] Garcia-Beltran, W. F. et al. Multiple SARS-CoV-2 variants escape neutralization by vaccine-induced humoral immunity. *Cell* **184**, 2372–2383.e9 (2021). 10.1016/j.cell.2021.03.013 PMC7953441 33743213

[19] Liu, K. et al. Binding and molecular basis of the bat coronavirus RaTG13 virus to ACE2 in humans and other species. *Cell* **184**, 3438–3451.e10 (2021). 10.1016/j.cell.2021.05.031 PMC8142884 34139177

[20] Han P (2022). Receptor binding and complex structures of human ACE2 to spike RBD from omicron and delta SARS-CoV-2. Cell.

[21] Mannar D (2022). SARS-CoV-2 Omicron variant: antibody evasion and cryo-EM structure of spike protein-ACE2 complex. Science.

[22] McCallum M (2022). Structural basis of SARS-CoV-2 Omicron immune evasion and receptor engagement. Science.

[23] Cui Z (2022). Structural and functional characterizations of infectivity and immune evasion of SARS-CoV-2 Omicron. Cell.

[24] Du P., Gao G. F., Wang Q. The mysterious origins of the Omicron variant of SARS-CoV-2. *Innovation* **3**, 100206 (2022). 10.1016/j.xinn.2022.100206 PMC8757324 35043101

[25] Kupferschmidt K (2021). Where did ‘weird’ Omicron come from?. Science.

[26] Wei C (2021). Evidence for a mouse origin of the SARS-CoV-2 Omicron variant. J. Genet. Genomics.

[27] Pan T (2021). Infection of wild-type mice by SARS-CoV-2 B.1.351 variant indicates a possible novel cross-species transmission route. Signal Transduct. Target Ther..

[28] Han P (2021). Molecular insights into receptor binding of recent emerging SARS-CoV-2 variants. Nat. Commun..

[29] Zahradník J (2021). SARS-CoV-2 variant prediction and antiviral drug design are enabled by RBD in vitro evolution. Nat. Microbiol..

[30] Chen L (2021). Emergence of Multiple SARS-CoV-2 Antibody escape variants in an immunocompromised host undergoing convalescent plasma treatment. mSphere.

[31] Guigon, A. et al. Emergence of Q493R mutation in SARS-CoV-2 spike protein during bamlanivimab/etesevimab treatment and resistance to viral clearance. *J. Infect.* **84**, 248–288 (2021). 10.1016/j.jinf.2021.08.033 PMC8381628 34437928

[32] Liu, K. et al. Cross-species recognition of SARS-CoV-2 to bat ACE2. *Proc. Natl. Acad. Sci. USA* **118**, e2020216118 (2021). 10.1073/pnas.2020216118 PMC7817217 33335073

[33] Sun S (2021). Characterization and structural basis of a lethal mouse-adapted SARS-CoV-2. Nat. Commun..

[34] Niu S (2021). Molecular basis of cross-species ACE2 interactions with SARS-CoV-2-like viruses of pangolin origin. EMBO J..

[35] Zhou, D. et al. Evidence of escape of SARS-CoV-2 variant B. 1.351 from natural and vaccine-induced sera. *Cell* **184**, 2348–2361.e6 (2021). 10.1016/j.cell.2021.02.037 PMC7901269 33730597

[36] Focosi D (2021). Emergence of SARS-COV-2 spike protein escape mutation Q493R after treatment for COVID-19. Emerg. Infect. Dis..

[37] Cao Y (2021). Omicron escapes the majority of existing SARS-CoV-2 neutralizing antibodies. Nature.

[38] Cele, S. et al. Omicron extensively but incompletely escapes Pfizer BNT162b2 neutralization. *Nature* **602**, 654–656 (2022). 10.1038/s41586-021-04387-1 PMC8866126 35016196

[39] Zhang, L. et al. The significant immune escape of pseudotyped SARS-CoV-2 Variant Omicron. *Emerg. Microbes Infect.* **11**, 1–5 (2022). 10.1080/22221751.2021.2017757 PMC8725892 34890524

[40] Dinnon KH (2020). A mouse-adapted model of SARS-CoV-2 to test COVID-19 countermeasures. Nature.

[41] Huang K (2021). Q493K and Q498H substitutions in spike promote adaptation of SARS-CoV-2 in mice. EBioMedicine.

[42] Wang J (2020). Mouse-adapted SARS-CoV-2 replicates efficiently in the upper and lower respiratory tract of BALB/c and C57BL/6J mice. Protein Cell.

[43] Guan Y (2003). Isolation and characterization of viruses related to the SARS coronavirus from animals in southern China. Science.

[44] Vandegrift, K. J. et al. Detection of SARS-CoV-2 Omicron variant (B.1.1.529) infection of white-tailed deer. *bioRxiv*10.1101/2022.02.04.479189 (2022).

[45] Zhang J (2021). Structural impact on SARS-CoV-2 spike protein by D614G substitution. Science.

[46] Yang, B. et al. SNX27 suppresses SARS-CoV-2 infection by inhibiting viral lysosome/late endosome entry. *Proc. Natl. Acad. Sci. USA* **119**, e2117576119 (2022). 10.1073/pnas.2117576119 PMC8794821 35022217

[47] Hsieh CL (2020). Structure-based design of prefusion-stabilized SARS-CoV-2 spikes. Science.

[48] Zheng SQ (2017). MotionCor2: anisotropic correction of beam-induced motion for improved cryo-electron microscopy. Nat. Methods.

[49] Rohou A, Grigorieff N (2015). CTFFIND4: Fast and accurate defocus estimation from electron micrographs. J. Struct. Biol..

[50] Punjani A, Rubinstein JL, Fleet DJ, Brubaker MA (2017). cryoSPARC: algorithms for rapid unsupervised cryo-EM structure determination. Nat. Methods.

[51] Bepler T (2019). Positive-unlabeled convolutional neural networks for particle picking in cryo-electron micrographs. Nat. Methods.

[52] Pettersen EF (2004). UCSF Chimera-a visualization system for exploratory research and analysis. J. Comput. Chem..

[53] Emsley P, Lohkamp B, Scott WG, Cowtan K (2010). Features and development of Coot. Acta Crystallogr. D: Biol. Crystallogr..

[54] Adams PD (2010). PHENIX: a comprehensive Python-based system for macromolecular structure solution. Acta Crystallogr. D: Biol. Crystallogr..

[55] Chen VB (2010). MolProbity: all-atom structure validation for macromolecular crystallography. Acta Crystallogr. D: Biol. Crystallogr..

[56] Otwinowski Z, Minor W (1997). Processing of X-ray diffraction data collected in oscillation mode. Methods Enzymol..

[57] Read RJ (2001). Pushing the boundaries of molecular replacement with maximum likelihood. Acta Crystallogr. D: Biol. Crystallogr..

