# Supplementary material for: Broader-species receptor binding and structural bases of Omicron SARS-CoV-2 to both mouse and palm-civet ACE2s
Source: Cell Discov. 2022 Jul 12;8:65. doi: 10.1038/s41421-022-00431-0 (PMC9274624; doi:10.1038/s41421-022-00431-0)
Supplement: Supplementary file 1 — Supplementary Information [file 41421_2022_431_MOESM1_ESM.pdf]

**Supplementary information**

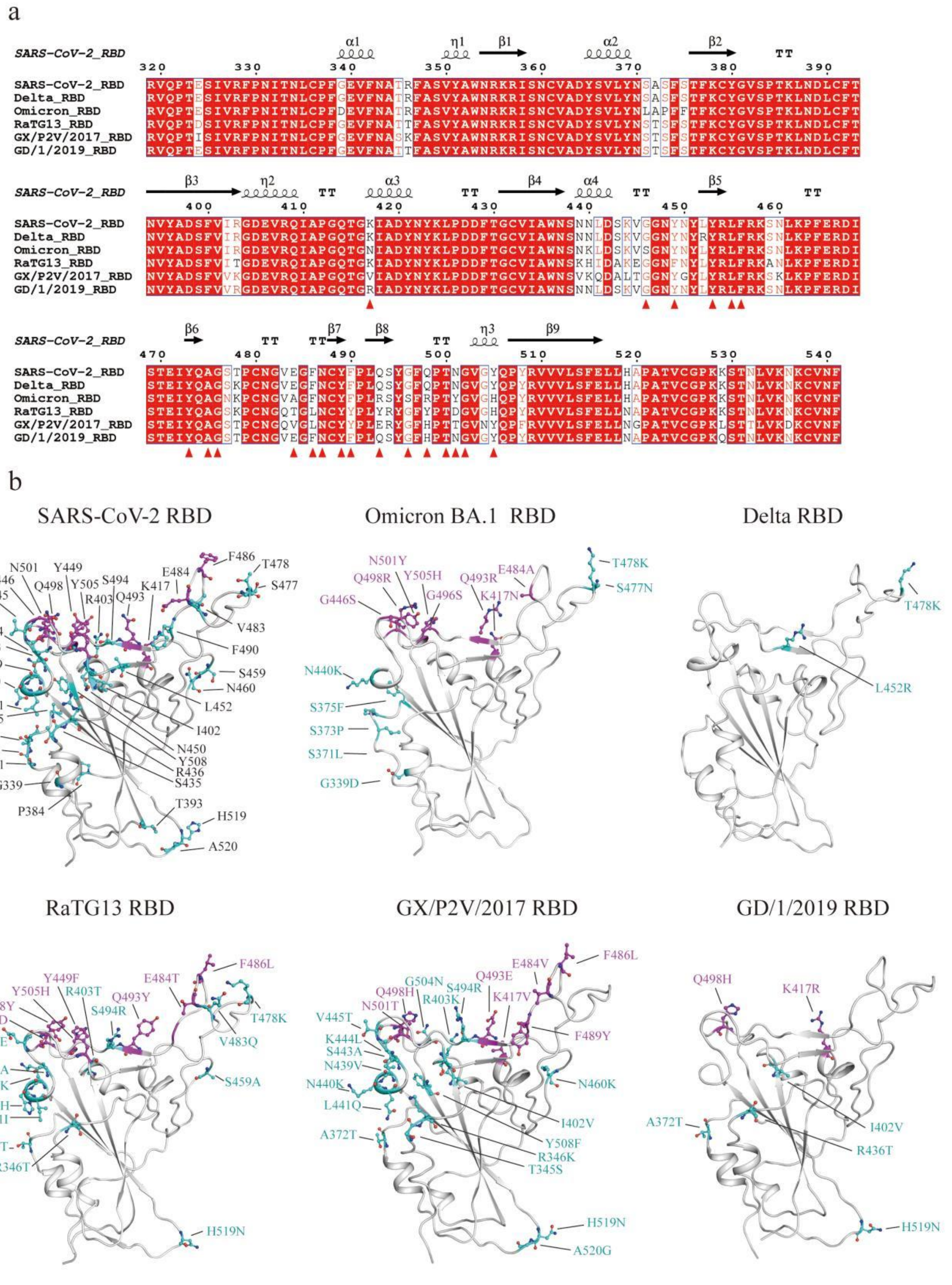


**Fig. S1 Sequence alignment and mutational mapping of RBDs from SARS-CoV-2 prototype, Omicron BA.1 variant, Delta variant, bat-origin RaTG13 and pangolin-origin GX/P2V/2017 and GD/1/2019.** (a) Sequence alignment of the six

RBDs. Residues of the prototype SARS-CoV-2 RBD interacting with hACE2 are labeled with red triangles. The alignment was performed by T-COFFEE and visualized by ESPript 3.0. (b) Mapping of substitutions on the six RBDs. Distinct residues of the corresponding RBD are represented as stick and balls. Residues located on or not on the hACE2 interface are colored in purple and cyan, respectively.

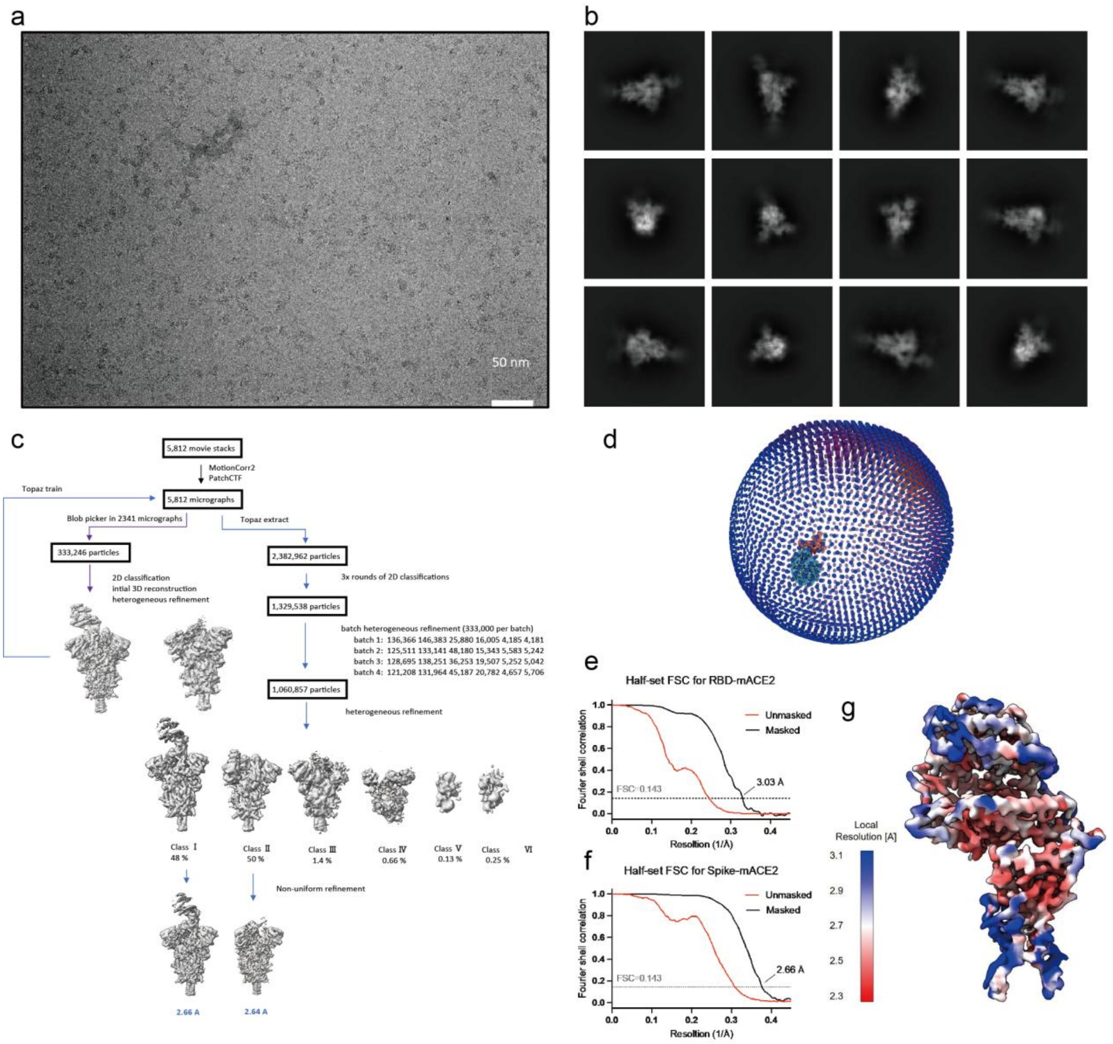


**Fig. S2 Cryo-EM data processing of the mACE2 and Omicron BA.1 S protein complex.** (a, b) Representative electron micrograph (a) and 2D class averages (b) of the mACE2 and Omicron BA.1 S protein embedded in vitreous ice. (c) Schematic to show the steps in cryo-EM data processing. (d) Angular distribution of the particles

for 3D reconstruction. (e) Fourier shell correlation (FSC) of the final EM map and model *vs.* the map for the mACE2 and Omicron BA.1 RBD complex. (f) Fourier shell correlation (FSC) of the final cryo-EM map and model *vs.* the map for the mACE2 and Omicron BA.1 S protein complex. (g) Local resolution map of the complex reconstruction.

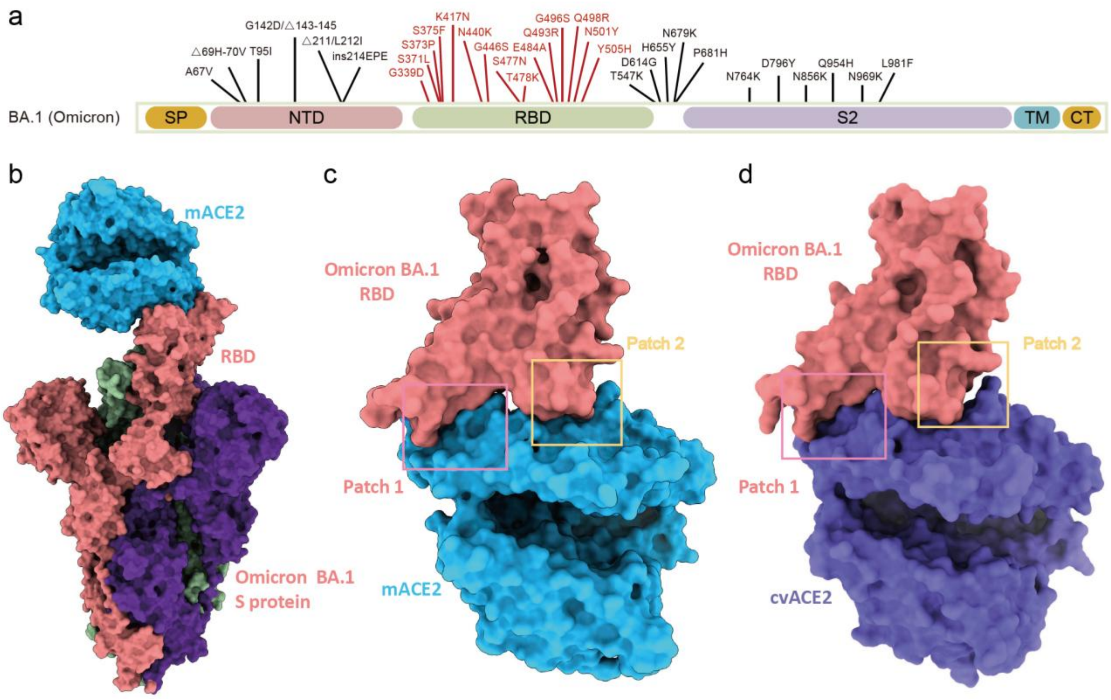


**Fig. S3 Mutation positioning of Omicron BA.1 S protein and overall structure of Omicron BA.1 S protein/mACE2 complex, Omicron BA.1 RBD/mACE2 and Omicron BA.1 RBD/cvACE2.** (a) The schematic diagram of the architecture of Omicron BA.1 S protein. The relative positioning of mutations is labeled. (b) Architecture of Omicron BA.1 S protein/mACE2 complex, Omicron BA.1 RBD/mACE2 (c) and Omicron BA.1 RBD/cvACE2 complexes (d). The boxes indicate clustered RBD residues interacting with ACE2.

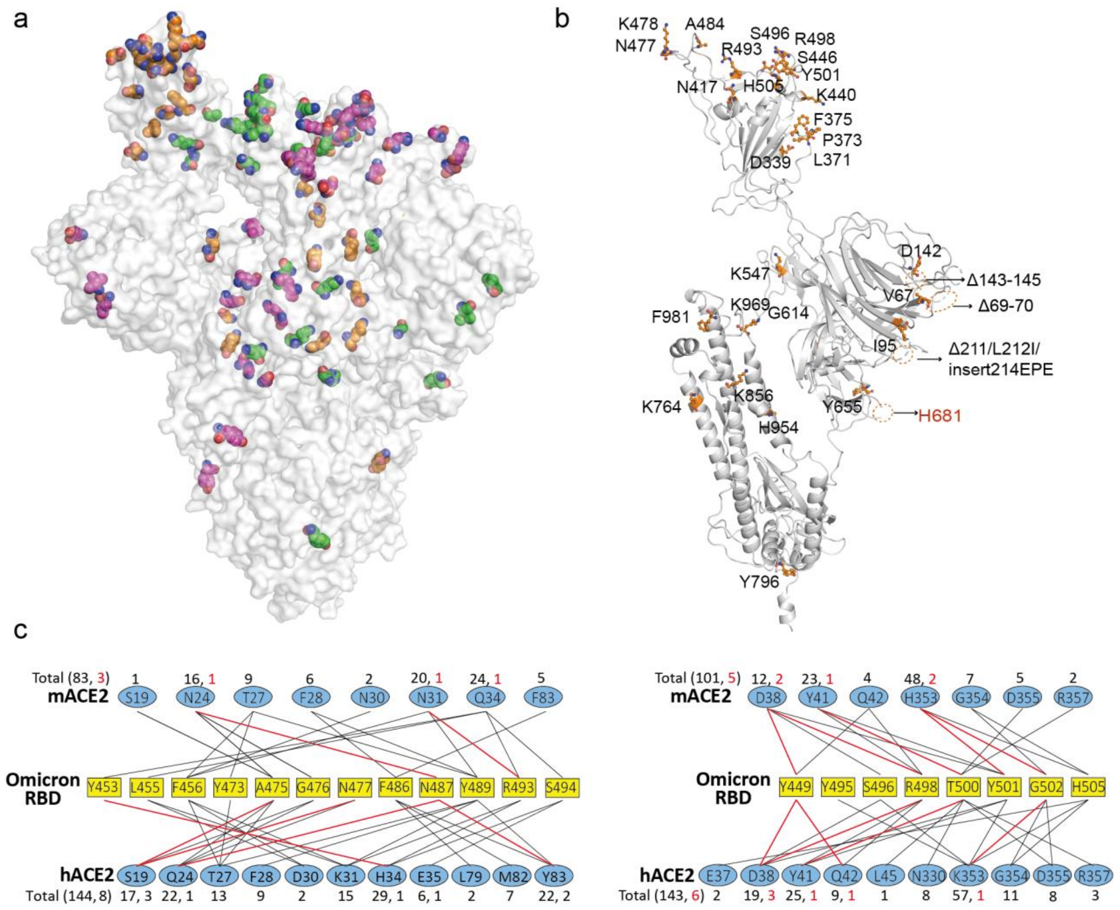


**Fig. S4 Mapping of observed mutations of the Omicron BA.1 S protein and interaction comparison between the Omicron BA.1 RBD and mACE2 or hACE2.** (a) Positioning of observed mutations are labeled on the S protein trimer; mutations on the three protomers are colored in orange, green and purple, respectively. (b) Mutation mapping of the Omicron S protein. Mutations are labeled with sticks and balls. (c) Residues involved in the interaction of hACE2 with the prototype RBD or Omicron RBD are listed and connected by solid lines. Black lines indicate van der Waals contacts, and red lines represent an H-bond or salt bridge.

| Species | 19 | 24 | 27 | 28 | 30 | 31 | 34 | 35 | 37 | 38 | 41 | 42 | 45 | 79 | 82 | 83 | 330 | 353 | 354 | 355 | 357 | Substitutions | |
|---|---|---|---|---|---|---|---|---|---|---|---|---|---|---|---|---|---|---|---|---|---|---|---|
| Human | S | Q | T | F | D | K | H | E | E | D | Y | Q | L | L | M | Y | N | K | G | D | R | | Primates |
| Monkey | S | Q | T | F | D | K | H | E | E | D | Y | Q | L | L | M | Y | N | K | G | D | R | 0 | |
| Rabbit | S | L | T | F | E | K | Q | E | E | D | Y | Q | L | L | T | Y | N | K | G | D | R | 4 | Lagomorpha |
| Guinea Pig | F | Q | T | F | D | E | L | K | E | D | Y | Q | L | L | A | Y | N | K | N | D | R | 6 | |
| Mouse | S | N | T | F | N | N | Q | E | E | D | Y | Q | L | T | S | F | N | H | G | D | R | 8 | Rodentia |
| Rat | S | K | S | F | N | K | Q | E | E | D | Y | Q | L | I | N | F | N | H | G | D | R | 8 | |
| Malayan pangolin | S | E | T | F | E | K | S | E | E | E | Y | Q | L | I | N | Y | N | K | H | D | R | 7 | Pholidota |
| Cat | S | L | T | F | E | K | H | E | E | E | Y | Q | L | L | T | Y | N | K | G | D | R | 4 | |
| Civet | S | L | T | F | E | T | Y | E | Q | E | Y | Q | V | L | T | Y | N | K | G | D | R | 8 | |
| Fox | S | L | T | F | E | K | Y | E | E | E | Y | Q | L | L | T | Y | N | K | G | D | R | 5 | |
| Dog | S | L | T | F | E | K | Y | E | E | E | Y | Q | L | L | T | Y | N | K | G | D | R | 5 | Carnivora |
| Raccoon dog | S | L | T | F | E | K | Y | E | E | E | Y | Q | L | L | T | Y | N | R | G | D | R | 6 | |
| Mink | S | L | T | F | E | K | Y | E | E | E | Y | Q | L | H | T | Y | N | K | R | D | R | 7 | |
| Horse | S | L | T | F | E | K | S | E | E | E | H | Q | L | L | T | Y | N | K | G | D | R | 6 | Perissodactyla |
| Pig | F | L | T | F | E | K | L | E | E | D | Y | Q | L | I | T | Y | N | K | G | D | R | 6 | |
| Wild Bactrian camel | S | L | T | F | E | E | H | E | E | D | Y | Q | L | T | T | Y | N | K | G | D | R | 5 | |
| Alpaca | S | L | T | F | K | E | H | E | E | D | Y | Q | L | A | T | Y | N | K | G | D | R | 5 | |
| Bovine | S | Q | T | F | E | K | H | E | E | D | Y | Q | L | M | T | Y | N | K | G | D | R | 3 | Artiodactyla |
| Goat | S | Q | T | F | E | K | H | E | E | D | Y | Q | L | M | T | Y | N | K | G | D | R | 3 | |
| Sheep | S | Q | T | F | E | K | H | E | E | D | Y | Q | L | M | T | Y | N | K | G | D | R | 3 | |
| Little brown bat | S | K | T | F | E | N | S | K | E | D | H | E | L | L | T | Y | N | K | G | D | R | 8 | |
| Fulvous fruit bat | S | L | T | F | E | K | T | E | E | D | Y | Q | L | L | T | Y | K | K | G | D | R | 5 | |
| Big-eared horseshoe bat | S | E | K | F | D | K | S | K | E | D | Y | E | L | L | N | Y | K | K | G | D | R | 7 | |
| Greater horseshoe bat | S | L | K | F | D | D | S | E | E | N | H | Q | L | L | N | F | N | K | G | D | R | 8 | Chiroptera |
| Chinese Horseshoe bat | S | E | I | F | D | K | T | K | E | D | H | Q | L | L | N | Y | N | K | G | D | R | 6 | |
| Least horseshoe bat | S | K | K | F | N | D | S | E | E | D | Y | Q | L | I | N | Y | N | K | G | D | R | 7 | |
| Lesser hedgehog tenrec | S | Q | S | F | T | T | N | E | E | N | Y | Q | L | L | K | F | K | L | N | D | R | 10 | Afrotheria |

**Fig. S5 Phylogenetic analysis of 26 animals based on ACE2 and characteristics of the SARS-CoV-2 prototype RBD-binding residues of ACE2s.** Twenty residues of hACE2 that are crucial in interacting with the SARS-CoV-2 prototype RBD are listed. Red letters indicate the substitutions in the ACE2 of 26 animal species.

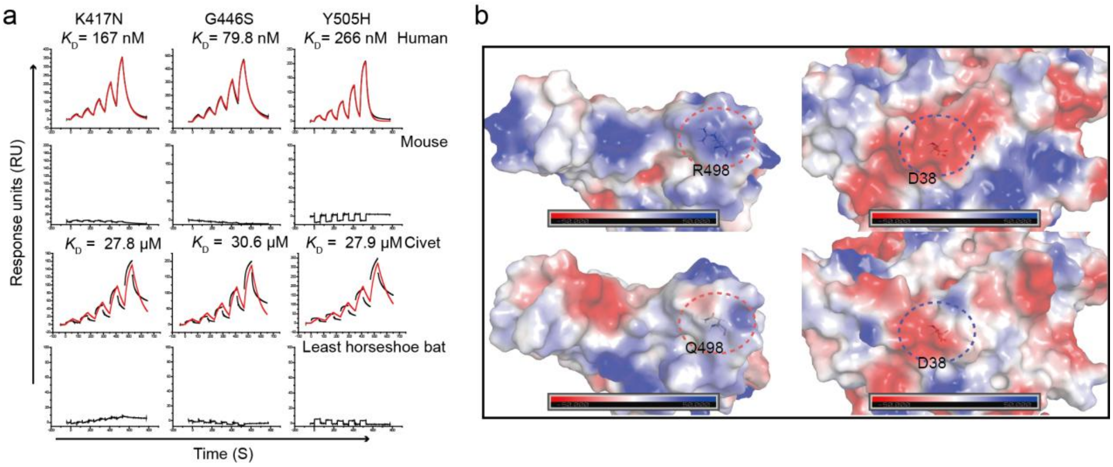


**Fig. S6 Effect on ACE2 binding of K417N, G446S and Y505H, and comparison of interaction network.** (a) SPR analysis of the binding affinity between mutated prototype RBD with ACE2s from human, mouse, civet, or least horseshoe bat, respectively. Raw and fitted curves are displayed as blue and red lines, respectively. (b)

The surface of Omicron BA.1 RBD (upper left), mACE2 (upper right), prototype RBD (lower left) and hACE2 (lower right) are colored for electrostatic potential: blue (basic), white (neutral), and red (acidic). Residues from hACE2 binding with the residue 498 of RBDs are circled with blue dotted ellipse, and residues R498 and Q498 of Omicron BA.1 RBD, and prototype RBD are circled with red dotted ellipse.

**Table S1 Cryo-EM data collection, refinement and validation statistics**

| | Omicron BA.1 S/mACE2 (PDB 7WRH) | Omicron BA.1 RBD /mACE2 (PDB 7WRI) |
|---|---|---|
| **Data collection and processing** | | |
| Magnification | 81k | 81k |
| Voltage (kV) | 300 | 300 |
| Electron exposure (e–/Å$^2$) | 60 | 60 |
| Defocus range (μm) | -1.0 ~ -2.0 | -1.0 ~ -2.0 |
| Pixel size (Å) | 1.11 | 1.11 |
| Symmetry imposed | C1 | C1 |
| Initial particle images (no.) | 2,382,962 | 2,382,962 |
| Final particle images (no.) | 503,967 | 503,967 |
| Map resolution (Å) | 2.66 | 3.03 |
| FSC threshold | 0.143 | 0.143 |
| | | |
| **Refinement** | | |
| Initial model used (PDB code) | 7XD7 | 6LZG |
| Model resolution range (Å) | Up to 3.3 | Up to 2.5 |
| Map sharpening *B* factor (Å$^2$) | 93.0 | 110.7 |
| Model composition | | |
| Non-hydrogen atoms | 30745 | 6431 |
| Protein residues | 3784 | 790 |
| Ligands | - | - |
| *B* factors (Å$^2$) | | |
| Protein | 30.7 | 47.3 |
| Ligand | 43.2 | 67.5 |
| R.m.s. deviations | | |
| Bond lengths (Å) | 0.002 | 0.003 |
| Bond angles (°) | 0.560 | 0.577 |
| Validation | | |
| MolProbity score | 1.65 | 2.00 |
| Clashscore | 5.71 | 6.44 |
| Poor rotamers (%) | 0.00 | 2.03 |
| Ramachandran plot | | |
| Favored (%) | 95.16 | 94.02 |
| Allowed (%) | 4.68 | 5.98 |
| Disallowed (%) | 0.16 | 0.00 |

**Table S2 Crystallographic data collection and refinement statistics.**

| | Omicron BA.1 RBD/cvACE2 |
|---|---|
| **Data collection** | |
| Space group | P 65 2 2 |
| Cell dimensions | |
| *a*, *b*, *c* (Å) | 152.67, 152.67, 187.11 |
| a, b, g (°) | 90.00, 90.00, 120.00 |
| Resolution (Å) | 50.00 - 3.30 (3.42 - 3.30) |
| $R_{merge}$ | 0.453 (1.549) |
| *I* / s*I* | 5.9 (1.5) |
| Completeness (%) | 99.7 (99.7) |
| Redundancy | 11.4 (11.1) |
| | |
| **Refinement** | |
| Resolution (Å) | 20.66-3.30 |
| No. reflections | 18538 |
| $R_{work}$ / $R_{free}$ | 0.2226/0.2752 |
| No. atoms | |
| Protein | 6418 |
| Ligand/ion | 96 |
| Water | 0 |
| *B*-factors | |
| Protein | 70.7 |
| Ligand/ion | 101.7 |
| Water | - |
| **R.m.s. deviations** | |
| Bond lengths (Å) | 0.004 |
| Bond angles (°) | 0.627 |
| **Ramachandran plot** | |
| Favored (%) | 94.16 |
| Allowed (%) | 5.34 |
| Outliers (%) | 0.51 |

*Values in parentheses are for highest-resolution shell.

**Table S3 Gene accession codes.**

| Genes | Accession codes | Databases |
|---|---|---|
| SARS-CoV-2 prototype RBD protein with his-tag, spike residues 319-541 | EPI_ISL_402119 | GISAID |
| SARS-CoV-2 Delta RBD protein with his-tag, spike residues 319-541 | EPI_ISL_2020954 | GISAID |
| SARS-CoV-2 Omicron BA.1 RBD protein with his-tag, spike residues 319-541 | EPI_ISL_6640916 | GISAID |
| Human ACE2 protein, residues 19-615 | NP_001358344 | GenBank |
| Mouse ACE2 protein, residues 19-615 | Q8R0I0 | UniProt |
| pEGFP-N1-hACE2 | BAJ21180 | GenBank |
| pEGFP-N1-Monkey ACE2 | A0A2K5X283 | UniProt |
| pEGFP-N1-Rabbit ACE2 | G1TEF4 | UniProt |
| pEGFP-N1-Guinea pig ACE2 | H0VSF6 | UniProt |
| pEGFP-N1-Mouse ACE2 | Q8R0I0 | UniProt |
| pEGFP-N1-Rat ACE2 | Q5EGZ1 | UniProt |
| pEGFP-N1-Malayan pangolin ACE2 | XP_017505746 | GenBank |
| pEGFP-N1-Cat ACE2 | Q56H28 | UniProt |
| pEGFP-N1-Civet ACE2 | Q56NL1.1 | UniProt |
| pEGFP-N1-Fox ACE2 | XP_025842512.1 | GenBank |
| pEGFP-N1-Dog ACE2 | J9P7Y2 | UniProt |
| pEGFP-N1-Raccoon dog ACE2 | ABW16956.1 | GenBank |
| pEGFP-N1-Horse ACE2 | F6V9L3 | UniProt |
| pEGFP-N1-Pig ACE2 | A0A220QT48 | UniProt |
| pEGFP-N1-wild Bactrian camel ACE2 | XP_006194263.1 | GenBank |
| pEGFP-N1-Alpaca ACE2 | XP_006212709.1 | GenBank |
| pEGFP-N1-Bovine ACE2 | Q58DD0 | UniProt |
| pEGFP-N1-Goat ACE2 | XP_005701129.2 | GenBank |
| pEGFP-N1-Sheep ACE2 | W5PSB6 | UniProt |
| pEGFP-N1-Little brown bat ACE2 | G1PXH7 | UniProt |
| pEGFP-N1-Fulvous fruit bat ACE2 | D8WU01 | UniProt |
| pEGFP-N1-Greater horseshoe bat ACE2 | B6ZGN7 | UniProt |
| pEGFP-N1-Chinese horseshoe bat ACE2 | E2DHI4 | UniProt |
| pEGFP-N1-Least horseshoe bat ACE2 | E2DHI9 | UniProt |
| pEGFP-N1-European hedgehog ACE2 | XP_007538670.1 | GenBank |
| pEGFP-N1-Lesser hedgehog tenrec ACE2 | XP_004710002.1 | GenBank |
| pEGFP-N1-Mink ACE2 | XP_044091953.1 | GenBank |

**Table S4 The immobilization and concentrations statistics of SPR assay to test the binding affinities of different ACE2 orthologs with prototype, Delta and Omicron BA.1 RBDs.**

| Ligand | Immobilization quantity(units) | Concentrations of prototype RBD (nM) | Concentrations of Delta RBD (nM) | Concentrations of Omicron BA.1 RBD (nM) |
|---|---|---|---|---|
| Human ACE2-mFc | 12616.30 | 200, 100, 50, 25, 12.5 | 200, 100, 50, 25, 12.5 | 200, 100, 50, 25, 12.5 |
| Macaca ACE2-mFc | 5517.52 | 100, 50, 25, 12.5, 6.25 | 100, 50, 25, 12.5, 6.25 | 100, 50, 25, 12.5, 6.25 |
| Rabbit ACE2-mFc | 9114.87 | 400, 200, 100, 50, 25 | 400, 200, 100, 50, 25 | 400, 200, 100, 50, 25 |
| Mouse ACE2-mFc | 7344.63 | 3200, 1600, 800, 400, 200 | 3200, 1600, 800, 400, 200 | 100, 50, 25, 12.5, 6.25 |
| Rat ACE2-mFc | 4213.01 | 3200, 1600, 800, 400, 200 | 3200, 1600, 800, 400, 200 | 3200, 1600, 800, 400, 200 |
| Malayan pangolin ACE2-mFc | 1859.69 | 800, 400, 200, 100, 50 | 800, 400, 200, 100, 50 | 3200, 1600, 800, 400, 200 |
| Cat ACE2-mFc | 8049.70 | 800, 400, 200, 100, 50 | 800, 400, 200, 100, 50 | 800, 400, 200, 100, 50 |
| Civet ACE2-mFc | 1769.34 | 32000, 16000, 8000, 4000, 2000 | 32000, 16000, 8000, 4000, 2000 | 3200, 1600, 800, 400, 200 |
| Fox ACE2-mFc | 3013.28 | 400, 200, 100, 50, 25 | 400, 200, 100, 50, 25 | 400, 200, 100, 50, 25 |
| Dog ACE2-mFc | 3099.70 | 800, 400, 200, 100, 50 | 800, 400, 200, 100, 50 | 800, 400, 200, 100, 50 |
| Raccon dog ACE2-mFc | 7089.21 | 800, 400, 200, 100, 50 | 800, 400, 200, 100, 50 | 800, 400, 200, 100, 50 |
| Horse ACE2-mFc | 4314.65 | 3200, 1600, 800, 400, 200 | 3200, 1600, 800, 400, 200 | 3200, 1600, 800, 400, 200 |
| Pig ACE2-mFc | 3426.59 | 800, 400, 200, 100, 50 | 800, 400, 200, 100, 50 | 800, 400, 200, 100, 50 |
| Wild bactrian camel ACE2-mFc | 3159.16 | 200, 100, 50, 25, 12.5 | 200, 100, 50, 25, 12.5 | 200, 100, 50, 25, 12.5 |
| Alpaca ACE2-mFc | 6781.92 | 400, 200, 100, 50, 25 | 400, 200, 100, 50, 25 | 400, 200, 100, 50, 25 |

| | | | | |
|---|---|---|---|---|
| Bovine ACE2-mFc | 15367.00 | 800, 400, 200, 100, 50 | 800, 400, 200, 100, 50 | 800, 400, 200, 100, 50 |
| Goat ACE2-mFc | 4725.20 | 800, 400, 200, 100, 50 | 800, 400, 200, 100, 50 | 800, 400, 200, 100, 50 |
| Sheep ACE2-mFc | 7213.00 | 800, 400, 200, 100, 50 | 800, 400, 200, 100, 50 | 800, 400, 200, 100, 50 |
| Intermediate horseshoe bat ACE2-mFc | 7582.10 | 800, 400, 200, 100, 50 | 800, 400, 200, 100, 50 | 800, 400, 200, 100, 50 |
| Little brown bat ACE2-mFc | 1190.77 | 800, 400, 200, 100, 50 | 800, 400, 200, 100, 50 | 12800, 6400, 3200, 1600, 800 |
| Fulvous fruit bat ACE2-mFc | 2067.08 | 800, 400, 200, 100, 50 | 800, 400, 200, 100, 50 | 12800, 6400, 3200, 1600, 800 |
| Greater horseshoe bat ACE2-mFc | 5985.45 | 3200, 1600, 800, 400, 200 | 3200, 1600, 800, 400, 200 | 6400, 3200, 1600, 800, 400 |
| Least horseshoe bat ACE2-mFc | 2601.83 | 3200, 1600, 800, 400, 200 | 3200, 1600, 800, 400, 200 | 12800, 6400, 3200, 1600, 800 |
| Big-eared horseshoe bat ACE2-mFc | 3159.16 | 3200, 1600, 800, 400, 200 | 3200, 1600, 800, 400, 200 | 3200, 1600, 800, 400, 200 |
| Lesser hedgehog tenrec ACE2-mFc | 7296.53 | 3200, 1600, 800, 400, 200 | 3200, 1600, 800, 400, 200 | 3200, 1600, 800, 400, 200 |
| Mink ACE2-mFc | 2338.53 | 800, 400, 200, 100, 50 | 800, 400, 200, 100, 50 | 800, 400, 200, 100, 50 |

**Table S5 The immobilization and concentrations statistics of SPR assays to test the binding affinities of the RBD substitutions with human, mouse, palm-civet and least horseshoe bat ACE2s.**

| Ligand | Immobilization quantity(units) | Concentrations of hACE2-His (nM) | Concentrations of mACE2-His (nM) | Concentrations of cvACE2-His (nM) | Concentrations of least horseshoe bat ACE2-His (nM) |
|---|---|---|---|---|---|
| SARS-CoV-2 prototype RBD | 2624.62 | 200, 100, 50, 25, 12.5 | 12800, 6400, 3200, 1600, 800 | 32000, 16000, 8000, 4000, 2000 | 12800, 6400, 3200, 1600, 800 |
| SARS-CoV-2 Omicron BA.1 RBD | 4038.68 | 200, 100, 50, 25, 12.5 | 200, 100, 50, 25, 12.5 | 3200, 1600, 800, 400, 200 | 6400, 3200, 1600, 800, 400 |
| SARS-CoV-2 prototype-K417N RBD | 2370.33 | 200, 100, 50, 25, 12.5 | 12800, 6400, 3200, 1600, 800 | 12800, 6400, 3200, 1600, 800 | 12800, 6400, 3200, 1600, 800 |
| SARS-CoV-2 prototype-G446S RBD | 1014.29 | 200, 100, 50, 25, 12.5 | 12800, 6400, 3200, 1600, 800 | 12800, 6400, 3200, 1600, 800 | 12800, 6400, 3200, 1600, 800 |
| SARS-CoV-2 prototype-S477N RBD | 2664.15 | 200, 100, 50, 25, 12.5 | 12800, 6400, 3200, 1600, 800 | 32000, 16000, 8000, 4000, 2000 | 12800, 6400, 3200, 1600, 800 |
| SARS-CoV-2 prototype-E484K RBD | 2773.66 | 200, 100, 50, 25, 12.5 | 12800, 6400, 3200, 1600, 800 | 32000, 16000, 8000, 4000, 2000 | 12800, 6400, 3200, 1600, 800 |

| | | | | | |
|---|---|---|---|---|---|
| SARS-CoV-2 prototype-Q493R RBD | 3448.97 | 200, 100, 50, 25, 12.5 | 32000, 16000, 8000, 4000, 2000 | 32000, 16000, 8000, 4000, 2000 | 32000, 16000, 8000, 4000, 2000 |
| SARS-CoV-2 prototype-G496S RBD | 3141.15 | 200, 100, 50, 25, 12.5 | 32000, 16000, 8000, 4000, 2000 | 12800, 6400, 3200, 1600, 800 | 12800, 6400, 3200, 1600, 800 |
| SARS-CoV-2 prototype-Q498R RBD | 3844.70 | 200, 100, 50, 25, 12.5 | 32000, 16000, 8000, 4000, 2000 | 32000, 16000, 8000, 4000, 2000 | 12800, 6400, 3200, 1600, 800 |
| SARS-CoV-2 prototype-N501Y RBD | 2915.73 | 200, 100, 50, 25, 12.5 | 32000, 16000, 8000, 4000, 2000 | 32000, 16000, 8000, 4000, 2000 | 32000, 16000, 8000, 4000, 2000 |
| SARS-CoV-2 prototype-Y505H RBD | 4600.96 | 200, 100, 50, 25, 12.5 | 12800, 6400, 3200, 1600, 800 | 12800, 6400, 3200, 1600, 800 | 12800, 6400, 3200, 1600, 800 |
